# Exploring glycolytic enzymes in disease: potential biomarkers and therapeutic targets in neurodegeneration, cancer and parasitic infections

**DOI:** 10.1098/rsob.240239

**Published:** 2025-02-05

**Authors:** Maura Rojas-Pirela, Diego Andrade-Alviárez, Verónica Rojas, Miguel Marcos, Daniel Salete-Granado, Marirene Chacón-Arnaude, María Á. Pérez-Nieto, Ulrike Kemmerling, Juan Luis Concepción, Paul A. M. Michels, Wilfredo Quiñones

**Affiliations:** ^1^ Instituto de Investigación Biomédica de Salamanca (IBSAL), Salamanca 37007, Spain; ^2^ Unidad de Medicina Molecular, Departamento de Medicina, Universidad de Salamanca, Salamanca 37007, Spain; ^3^ Servicio de Medicina Interna, Hospital Universitario de Salamanca, Salamanca 37007, Spain; ^4^ Laboratorio de Enzimología de Parásitos, Departamento de Biología, Facultad de Ciencias, Universidad de Los Andes, Mérida 5101, Venezuela; ^5^ Instituto de Biología, Facultad de Ciencias, Pontificia Universidad Católica de Valparaíso, Valparaíso 2373223, Chile; ^6^ Fundación Instituto de Estudios de Ciencias de la Salud de Castilla y León, Soria 42002, Spain; ^7^ Instituto de Ciencias Biomédicas, Universidad de Chile, Facultad de Medicina, Santiago de Chile 8380453, Chile; ^8^ School of Biological Sciences, University of Edinburgh, The King’s Buildings, Edinburgh EH9 3FL, UK

**Keywords:** glycolysis, pathogenesis of diseases, prognostic, diagnostic, therapeutic

## Abstract

Glycolysis, present in most organisms, is evolutionarily one of the oldest metabolic pathways. It has great relevance at a physiological level because it is responsible for generating ATP in the cell through the conversion of glucose into pyruvate and reducing nicotinamide adenine dinucleotide (NADH) (that may be fed into the electron chain in the mitochondria to produce additional ATP by oxidative phosphorylation), as well as for producing intermediates that can serve as substrates for other metabolic processes. Glycolysis takes place through 10 consecutive chemical reactions, each of which is catalysed by a specific enzyme. Although energy transduction by glucose metabolism is the main function of this pathway, involvement in virulence, growth, pathogen–host interactions, immunomodulation and adaptation to environmental conditions are other functions attributed to this metabolic pathway. In humans, where glycolysis occurs mainly in the cytosol, the mislocalization of some glycolytic enzymes in various other subcellular locations, as well as alterations in their expression and regulation, has been associated with the development and progression of various diseases. In this review, we describe the role of glycolytic enzymes in the pathogenesis of diseases of clinical interest. In addition, the potential role of these enzymes as targets for drug development and their potential for use as diagnostic and prognostic markers of some pathologies are also discussed.

**Figure:**
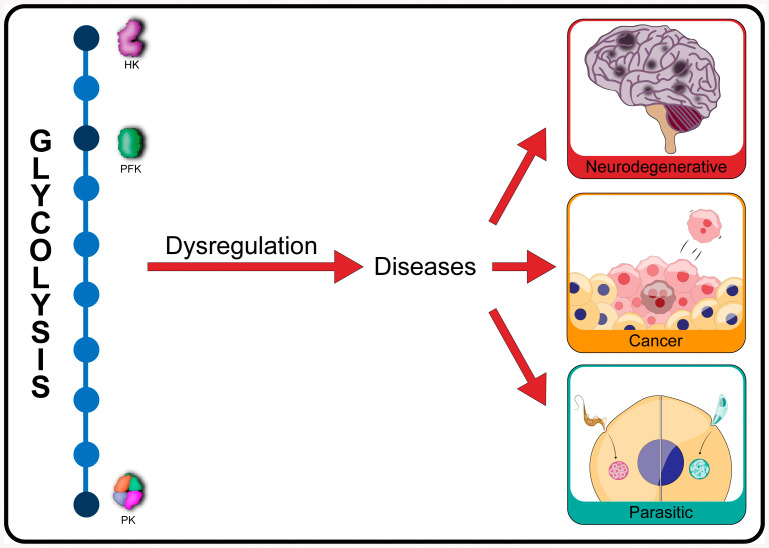

## 1. Introduction

Metabolism is a fundamental process in all living organisms and consists of a network of biochemical reactions that transform metabolites to fulfil biological functions [1]. The reactions may be divided into those involved in breakdown of complex molecules, yielding a biological useful form of energy—most often ATP (catabolism: demand exceeds supply), and reactions for the synthesis of complex molecules with the involvement of energy storage (anabolism: supply exceeds demand) [2]. These reactions are catalysed and regulated by a wide variety of enzymes, which support many distinct cellular processes, such as growth and proliferation, involving the synthesis of cellular components and generation of forms of energy to sustain them [3].

Generally, these enzymes act in an organized manner within a metabolic pathway, in which the products of one enzyme can function as a substrate for the next step of the pathway [2]. In eukaryotic cells, functional specialization of metabolic pathways is conditioned by compartmentalization. Intermediary metabolism (all reactions involved in producing and using metabolic energy for the biosynthesis of low-molecular-weight compounds and energy storage compounds) occurs in the cytosol and other subcellular locations (e.g. mitochondria), whereas genome replication, maintenance and expression are mainly carried out in the nucleus [4]. However, cytoplasmic (i.e. cytosolic and organellar) metabolism has a great influence on the processes that occur in the nucleus, through the activation of transcription factors by metabolic sensors, rendering available cofactors of chromatin-modifying enzymes, and the activity of specialized metabolic enzymes which can modulate chromatin structure and gene expression, establishing a coordination of metabolism and gene expression [4]. A relationship between metabolism and epigenetic inheritance has been suggested, where nutritional status modulates gene expression patterns that involve changes in chromatin structure and the expression of small non-coding RNAs [4,5].

There are numerous metabolic pathways, and a significant part of them is registered in specialized databases such as the Kyoto Encyclopedia of Genes and Genomes (KEGG) [6] and MetaCyc Metabolic Pathway Database [7]. As of 2023, MetaCyc contained approximately 3100 metabolic pathways and 18 500 biochemical reactions [8]. In humans, the most-studied metabolic pathways are the tricarboxylic acid (TCA) cycle (also known as citric acid cycle and Krebs cycle), oxidative phosphorylation (OXPHOS), urea cycle, fatty-acid β-oxidation, pentose-phosphate pathway (PPP) and the pathways for gluconeogenesis and glycolysis. The regulation of these pathways is critical for cellular homeostasis; alterations in each of these pathways are observed in a wide variety of pathological processes [2,9–13]. Abnormalities in processes such as glycolysis have been associated with various disorders such as neurodegenerative [14], metabolic [15], infectious [16], autoimmune and inflammatory [17,18] diseases, as well as cancer [19]; glycolytic enzymes have been proposed as potential biomarkers and/or targets for the diagnosis, treatment and prognosis of many of these diseases. Some strategies, such as gene therapy, targeted therapy and immunotherapy, may act as novel therapeutic approaches through application on glycolysis in the treatment of these pathologies [20,21]. In this review, we will focus on glycolytic enzymes, emphasizing the possible role that their alterations have in the development of some pathologies and the potential use of glycolytic enzymes as chemotherapeutic targets and biomarkers for the diagnosis and prognosis of various diseases.

## 2. Glycolysis and its enzymes

In addition to the glycolytic or Embden–Meyerhof–Parnas (EMP) pathway, which is widely distributed across all three domains of nature [22], another route exists to convert glucose into pyruvate, the Entner–Douderoff (ED) pathway where (when its reactions are linked to the lower trunk of the EMP pathway) one molecule of glucose yields two pyruvate molecules and one molecule of ATP, NADH and NADPH [23]. However, this ED pathway is only found in prokaryotes. In addition, glucose is also metabolized via the PPP that does not yield any ATP but is a major contributor of NADPH to cells [24].

**Figure 1.**
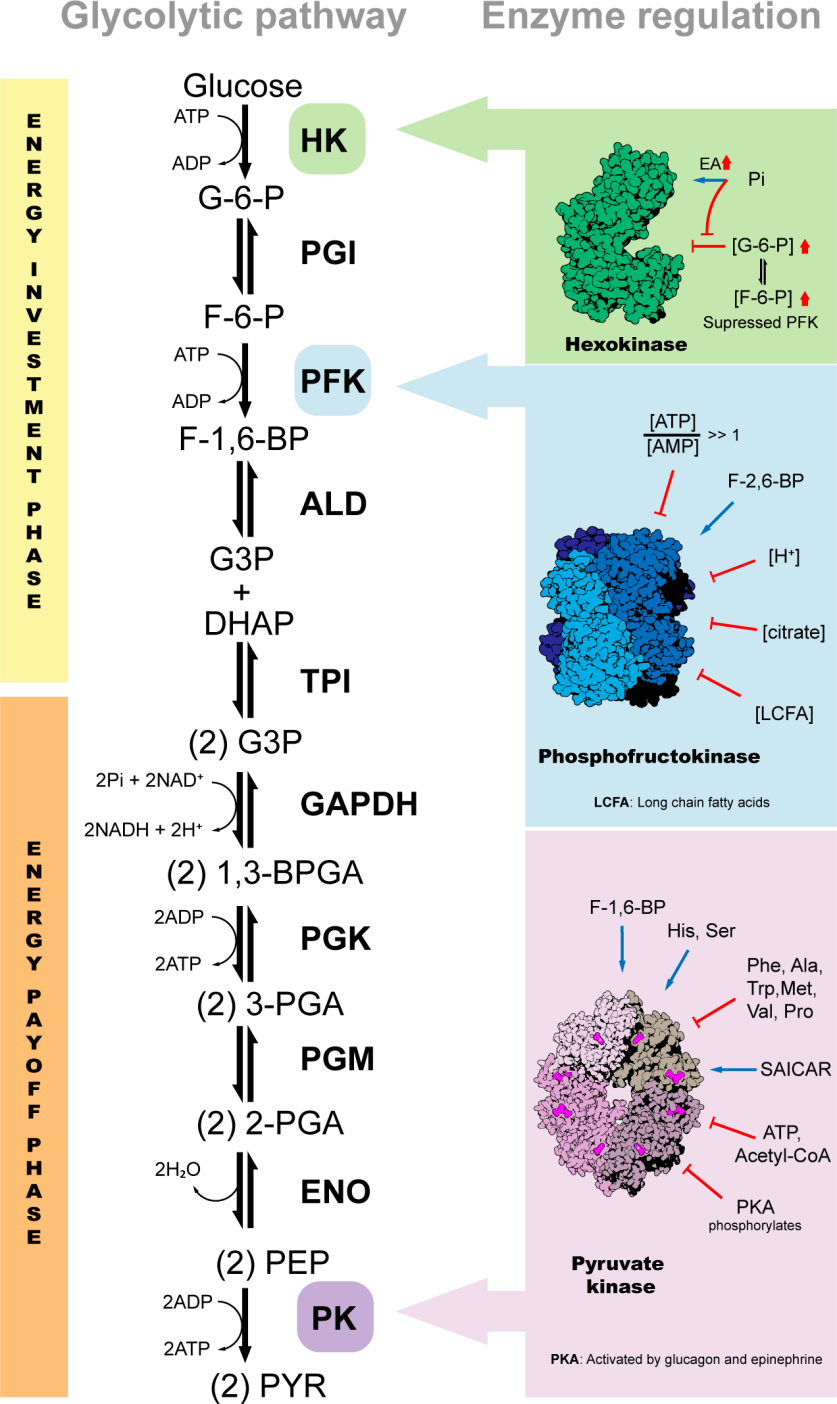
Glycolytic pathway. This pathway contains 10 enzymatically catalysed steps and is divided into two phases, the investment phase (in which two molecules of ATP are consumed, in the reactions by HK and PFK) and the energy payment phase (where two molecules of ATP are generated, in the reactions by PGK and PYK), respectively. The pathway is regulated by the three enzymes which catalyse the reactions which, under physiological conditions, are essentially irreversible, HK, PFK and PK, through modulation of their activities by different metabolites. HK is regulated mainly by intermediates of the glycolytic pathway; it is negatively modulated by its product G-6-P and the PFK product F-6-P. Molecules such as orthophosphate (Pi) can also influence HK activity; its binding to the N-terminal part of this enzyme suppresses the inhibitory effect caused by G-6-P. For their part, PFK and PK are regulated by metabolites of different nature. PFK activity is modulated ATP, F-2,6-BP, pH; as well as by molecules such as fatty acids, and carboxylic acids (notably citrate), while PK can be modulated by PFK's product F-1,6BP, amino acids (Phe, Ala, Trp, Met, Val, Pro, His and Ser), non-glycolytic metabolites (e.g. succinyl-5-aminoimidazole-4-carboxamide-1-ribose-5′-phosphate (SAICAR)), ATP, acetyl-CoA and by post-translational modification by PKA.

The EMP pathway is a highly conserved metabolic pathway that converts glucose into pyruvate, thereby generating ATP, NADH and intermediates that can act as precursors for various biosynthetic processes (such as glucose-6-phosphate (G-6-P) and dihydroxyacetone phosphate (DHAP)) [25]. Two ATP and two NADH molecules are produced for each glucose molecule that is broken down. The generated pyruvate molecules may subsequently be completely metabolized to CO_2_ in the mitochondrial TCA cycle and the NADH reoxidized by respiration coupled to additional ATP synthesis by OXPHOS [26]. This EMP pathway is made up of 10 steps of chemical reactions, each catalysed by a specific enzyme (figure 1), which are hexokinase (HK), phosphoglucose isomerase (PGI), phosphofructokinase (PFK), aldolase (ALD), triosephosphate isomerase (TPI), glyceraldehyde-3-phosphate dehydrogenase (GAPDH), phosphoglycerate kinase (PGK), phosphoglycerate mutase (PGM), enolase (ENO) and pyruvate kinase (PK) [27]. Whereas in the upper part of glycolysis (UG) the glucose molecules are activated for catabolism at the expense of ATP in two phosphorylation reactions catalysed by HK and PFK, formation of four molecules of ATP occurs in the lower part of glycolysis (LG), in reactions catalysed by PGK and PK, thus producing a net yield of two ATP molecules per glucose consumed [27] (figure 1). This organization of the pathway represents a so-called ‘turbo design’ implying a requirement for ATP hydrolysis in the initial part of the pathway (investment phase), followed by a surplus ATP generation downstream in the process (yield or pay-off phase). The net yield of ATP produced along the pathway will activate the initial ATP-requiring reactions, a potentially dangerous design necessitating the regulation of the activity of the enzymes involved by their products to keep the process in check [28].

One of the characteristics of glycolysis is that it can occur both in the absence of oxygen (anaerobically) and in the presence of oxygen (aerobically). Under anaerobic conditions, the final product is lactate (or ethanol in some prokaryotes), resulting from the reoxidation of NADH with pyruvate as substrate, while under aerobic conditions, the pyruvate may be further oxidized to CO_2_ and H_2_O in the mitochondria [29]. However, in cells that lack mitochondria, such as erythrocytes [30], glycolysis represents the main source of ATP synthesis [29]. Cells of some organs such as kidneys, gastrointestinal tract, retina, skin and brain also derive most of their energy from glycolysis [29]. In the case of the brain, glycolysis is very important, since it participates in various neural activities, through ATP generation and redox homeostasis [26]. Additionally, some studies suggest that, from a whole-brain perspective, aerobic glycolysis may account for 10–12% of glucose used in adult humans [31,32]. However, this percentage can vary between adults and newborns. In the latter, it can amount up to 30% of metabolized glucose [33].

Control of the glycolytic flux plays an important role in the physiology of mammals [34], not only due to the production of NADH and ATP to satisfy the energy needs of the cell, but also the generation of some intermediates for other metabolic pathways such as for the de novo synthesis of phospholipids, triglycerides, fatty acids and amino acids [27], as well as the production of other biomass building blocks in the contexts of processes such as cell proliferation and angiogenesis [26,34]. Dysregulation of glycolysis is a hallmark of diseases such as cancer [35,36], a topic that will be discussed in following sections.

### 2.1. Origin and evolution of glycolysis

The emergence and evolution of metabolism have been fundamental in the evolutionary development of organisms, since it not only allowed organisms to become less dependent on exogenous sources of organic compounds but also influenced adaptation to the environment, by regulation of the internal environment as well as establishing ecological interactions [37,38]. Although the timing of metabolic pathway origination is poorly understood, it is believed to have taken place approximately 3.7 billion years ago [39] when many key reactions of some metabolic pathways may have occurred through a primitive non-enzymatic version of metabolism (protometabolism), initially catalysed by naturally occurring mineral surfaces, metal or other inorganic ions, as well as small organic molecules [40–42]. They may have originated in specialized extreme environments, such as hydrothermal vents at fissures in the ocean floor or in fluctuating hot spring pools at volcanic surfaces, which offered ideal conditions, including high temperatures and minerals, for the formation of biomolecules and the synthesis of precursors, such as simple organic molecules, amino acids, nucleotides and lipids, necessary to support emerging primitive life forms [43–45]. It is feasible that the universal core of metabolism arose from prebiotic pathways favoured both thermodynamically and kinetically in the origins of life [46,47]. In fact, some studies have shown that some parts of the central metabolic pathways, including glycolysis and the TCA cycle, can occur spontaneously without enzymes [42,48,49]. The transition from protometabolism to modern (enzymatic) metabolism could have involved the emergence of catalysts through a selection process that would have been based primarily on their utility under specific conditions [42].

In those hydrothermal outlets, lipid membranes could have formed spontaneously, since both the fluctuations in the temperature and chemical composition of the volcanic pools and the salinity and alkaline conditions in the oceanic vents could have generated concentration gradients that influenced the formation of membranous structures or protocellular structures [44,50,51]. Additionally, a theoretical model which considers the synthesis of nucleotides via a branching protometabolism [46], based on universally conserved pathways [47], suggests that some nucleotides could have been formed at trace levels within these protocells and favoured the growth and replication of the protocells by simple means [46]. The most striking aspect about this model is that it proposes a scenario for the appearance of the molecules necessary for the origin of the genetic code and, consequently, the emergence of genetic inheritance [46]. Importantly, the evolution of transport systems through cell membranes had a relevant impact on the early evolution of life. The separation of substances through active transport systems in the simplest cells could have facilitated the uptake of nutrients and the elimination of waste by protocells [52], which would have been fundamental for their survival and replication. The latter was what could have promoted the transition from protocells to the last universal common ancestor (LUCA).

Taking into account studies based on comparative physiology, which identified approximately 400 biosynthetic reactions commonly used by bacteria and archaea [47] that one could think of as universal reactions representing the central biosynthetic metabolism in the LUCA, this hypothetical ancestor could be seen as representing the end point of metabolic origin, and at the same time, the starting point of physiological diversification [47]

Various theories have tried to explain the emergence of metabolic pathways [53,54]. Some of these theories are based on Darwinian processes that imply the pre-existence of high biochemical complexity, while others take into account entirely non-enzymatic routes such as those that existed in the initial times of the origin of life [41,55–60]. Scenarios may have involved the emergence of an enzyme catalysing its synthesis from a compound already existing in the surrounding environment (*retrograde evolution*) [55], or oppositely, that metabolic pathways may have evolved in a ‘forward’ direction, from simple precursors to final products. In this latter case, the pathways would have originated from simple reactions and then have subsequently expanded and become more complex as organisms evolved and adapted to their environment (*forward evolution*) [56]. Alternatively, metabolic pathways may have been assembled through the recruitment of promiscuous primitive enzymes that could react with various chemically related substrates, leading to the formation of different end products, followed by development from being general enzymes with low substrate specificity to becoming specialists (patchwork hypothesis) [57,58]. Or, such promiscuous enzymes could have been part of a mosaic assembly, where they were able to catalyse different reactions in multiple pathways, but in turn were recruited to different metabolic pathways and subsequently developed specificity relevant for each distinct pathway [61]. Further, the evolution of metabolism may have undergone successive addition of different metabolic pathways, where a central pathway would precede other pathways added later. As more metabolic pathways were incorporated, the more internal pathways persist as a vestige of an older metabolism (shell hypothesis) [62]. In *enzyme–metabolite coevolution*, the secondary products (known as ‘underground metabolism’) of promiscuous enzymes or non-enzymatic reactions could serve as evolutionary stepping stones for the appearance of specialized enzymes that synthesize these products [60], while in the *protometabolic theory* enzymes would appear as a result of the need to decrease energy dissipation of protometabolism, by accelerating or increasing the specificity of reactions that benefit the network’s persistence [40,63,64]. Additionally, horizontal gene transfer (HGT) can contribute to evolution of metabolism in prokaryotes and unicellular eukaryotes, by transferring a promiscuous enzyme-coding gene, which can duplicate and diversify in the recipient organism [65].

Studies have documented the presence of glycolysis in the different major phylogenetic taxons of each of the three domains of the tree of life [66–71]. It is, therefore, considered to have appeared already early in the evolution of prokaryotes and also became present in the early eukaryotes, where it has a bacterial origin [72]. It is conceivable that the glycolytic pathway could have arisen from that of the reverse process, gluconeogenesis, after the establishment of some other universal metabolic routes such as those of the catabolism and anabolism of amino acids, metabolism of fatty acid-like molecules and the TCA cycle [73].

LUCA, the hypothetical common ancestral cell from which the three domains of life, the Bacteria, Archaea and Eukarya, originated, was likely an anaerobic thermophile in which pathways of non-oxidative metabolism of sugars were active, mainly associated with the synthesis of the primitive cell wall [69,74,75]. It has been suggested that LUCA was a more complex cell than often expected [75] and was capable of biochemistry like that observed in some anaerobic thermophilic organisms today [41,69]. This implies that cellular complexity arose near the very beginning of life and its metabolism has changed little over time with respect to extant anaerobic thermophilic archaea [41,69]. This conclusion is drawn from predictions of the LUCA protein family, based on a comparative analysis of genomic and proteomic data across the tree of life: 169 ancestral enzyme functions were identified in LUCA’s core proteome, many of them converging on metabolic categories such as those involving sugars (glycolysis/gluconeogenesis), amino acids, nucleotides, protein synthesis and use of nucleotide-derived cofactors [76]. Likewise, recent studies based on phylogenetic analyses, which reconstructed the genomic and metabolic characteristics of LUCA, have demonstrated that this organism was capable of gluconeogenesis and glycolysis. Most of the enzyme subunits involved in these pathways were identified. Additionally, gluconeogenesis may have been a relevant pathway for linking carbon fixation to nucleotide biosynthesis via the PPP, since many of the enzymes participating in this pathway also appear to have been present in LUCA [77]. All of this suggests a conserved genomic core, inherited by organisms across the tree of life, and furthermore that LUCA could represent a complex stage of early evolution [76].

Similarly, the last bacterial common ancestor (LBCA), the ancestor for the domain Bacteria, is considered to have been anaerobic and is predicted to have had approximately 2717 genes [75], some of them coding for at least 10 families of proteins related to carbohydrate and energy metabolism [71]. It must have contained glycolytic enzymes such as TPI, GAPDH, PGK, ENO and PK [71,78]. A recent pangenomic study has suggested a versatile core genome for LBCA, containing many complete or nearly complete biological systems, especially those related to ribosomal proteins, translation machinery and metabolic pathways of central carbon metabolism (such glycolysis, PPP and TCA cycle) and de novo biosynthetic pathways for amino acids, nucleotides and dNTPs [79]. In the case of glycolysis, the presence of genes encoding several enzymes related to glycolysis is suggested, additional to those previously reported [71], such as glucokinase (GLK), PGI, PFK, ALD, TPI, GAPDH, PGK, PGM, ENO and PK. Additionally, a phosphoenolpyruvate synthetase (PPSA) gene may have been present in the LBCA genome. This PPSA enzyme catalyses the conversion of pyruvate and ATP to phosphoenolpyruvate (PEP), AMP and inorganic phosphate, a reaction that functions in gluconeogenesis [80]. The absence of some enzymes, such as alanine dehydrogenase (ADH) and alanine transaminase (ALA), involved in direct conversion from pyruvate to alanine and in the glyoxylate cycle, is thought to provide some clues about its contemporary environment, i.e. an origin in hydrothermal vents [79].

Regarding the last archaeal common ancestor (LACA), computational studies have suggested that it was an anaerobic autotroph, able to produce methane in an anaerobic environment [71,81,82] and also to reduce CO_2_ to acetyl-CoA via the Wood–Ljungdahl pathway and subsequently to generate acetate and ATP using an acetyl-CoA synthetase [74,83]. Different from what has been predicted for LBCA, some authors suggested that most glycolytic enzymes were absent from LACA; only the PK would have been present in this organism. In addition, it probably contained a bifunctional fructose-1,6-bisphosphate aldolase/phosphatase (FBPA/FBPase) [74], a thermostable enzyme that catalyses two subsequent steps in gluconeogenesis in most archaea and deeply branched bacterial groups [84] and is considered to represent a pace-making ancestral gluconeogenic enzyme [85].

Importantly, a hypothetical first eukaryotic common ancestor (FECA), the first descendant of the last common ancestral node of eukaryotes and an ‘archaeal’, must have possessed many structural features of modern eukaryotes [68]. This common ancestral node is the point of emergence of the eukaryotic cell from the domain of archaeal life and represents the link between prokaryotes and eukaryotes [68,86]. This archaeal organism involved in the node belonged to the superphylum called ‘Asgard’ that includes related members forming a monophyletic group with eukaryotes in phylogenomic analyses [87,88]. These Asgards are characterized by having genomes that encode a wide repertoire of proteins previously assumed to be specific of eukaryotes (including homologues of eukaryotic proteins involved in cytoskeletal functions, membrane trafficking and vesicle formation/trafficking). Furthermore, Asgard genomes apparently encode a large number of proteins similar to bacterial proteins, which may explain the presence of bacterial genes in eukaryotic genomes [86,87].

Many models attempted to explain the origin of eukaryotes, but the lack of Asgard archaea in culture prevented the validation of these models [89]. However, experimental studies of an Asgard archaeon from the Lokiarchaeota phylum, called ‘*Prometheoarchaeum syntrophicum*’ [90], support models in which the eukaryotic cell could have evolved from an integrated archaeal–bacterial syntrophic consortium [90]. This has led to the proposal of a the Entangle–Engulf–Endogenize (E3) model, which involves three partners. In this model, a bacterium from the Alphaproteobacteria class, which eventually gave rise to mitochondria, was absorbed by an H_2_-producing Asgard archaeon that initially associated with a sulfate-reducing Deltaproteobacterium. However, this hydrogen-scavenging bacterial partner would have been lost in favour of the aerobic alphaproteobacterium that allowed aerotolerance through detoxifying oxygen [90,91]. During absorption, the archaeal host shared amino acid-derived metabolites with the endosymbiont which, in return, breathed oxygen and provided the host with building blocks that it might not synthesize [89]. Additional steps, such as acquiring an ATP transporter by the oxygen-respiring ATP-generating endosymbiont and the replacement of archaeal membrane lipids by bacterial ones, helped to convert this consortium into a eukaryotic cell [89,90]. It should be noted that some authors have discussed new perspectives, approaches or interpretations of the syntrophy hypothesis, presenting new ideas that complement or expand the original model [92].

Although the relative location and contribution of the endosymbiont and, consequently, the cellular complexity of the archaeal host prior to endosymbiosis have been debated [93], several studies support the idea that the endosymbiosis process appeared to be relevant for the transition from FECA to the last eukaryotic common ancestor (LECA) [89,94]. This FECA–LECA process was marked by a series of events that led to the acquisition of characteristics of modern eukaryotes (such as the cell nucleus, organelles, cytoskeleton and more complex metabolic systems) [67,93]. Eventually, a series of events occurred that resulted in the extinction of transitional eukaryotic forms and the radiation of LECA [68], which was characterized as a facultative anaerobe like a modern protist [92,95]. A metabolic reconstruction showed that LECA had the metabolic capacity to process glucose to pyruvate, oxidize acetate to CO_2_ and water, and then produce ATP from NADH via electron transport and OXPHOS, as do many modern eukaryotes [95].

Pathways such as those for glycolysis have likely undergone significant changes by HGTs and gene displacements post-LECA, notably during the evolution of anaerobic/microaerophilic lineages [67]. In most eukaryotes, glycolysis is catalysed by orthologues of classic Embden–Meyerhof glycolytic enzymes, and not the variants present in some archaea [96,97]. Additionally, as we will see later, other innovations related to this catabolic pathway have been observed in some protists and fungi, in which this pathway is partially compartmentalized in organelles such as mitochondria, peroxisomes and peroxisome-related organelles known as glycosomes because of the presence of glycolytic enzymes in their matrix [72,98–101].

### 2.2. Regulation of glycolysis in humans

In humans, the constituent enzymes of the glycolytic pathway manifest as distinct isoenzymes, exhibiting differential expression patterns in tissues throughout development. The isoenzyme numbers for these glycolytic enzymes vary between two and five. For certain enzymes, such as PFK, three isoenzymes have been identified, while for others, such as HK and PK, the existence of five and four isoenzymes has been documented [102–110]. The genes that code for these enzymes are concentrated on specific chromosomes [1,2,5,7,10,12,15,21], in some cases tandemly arranged [111]. In our preliminary studies of the genes coding for some glycolytic enzymes, we observed that alternative splicing of their mRNAs can result in multiple transcript variants. For example, for HK2, up to 8 variants can be generated, while for the enzymes PFKM and PKM, up to 18 and 40 variants, respectively, can be reached [111]. This is similar to what has been reported for other enzymes such as PGI, showing up to 24 variants in peripheral blood mononuclear cells, for which a mechanism of post-transcriptional modification has been suggested [112]. It is possible to think that the expression of these variants may have a relationship with the appearance or not of some diseases such as cancer. Above all, this is supported taking into account that other glycolytic isoenzymes, such as ENO-3, for which at least seven alternative mRNAs can be generated [111], are considered predictive of poor prognosis in patients [113,114]. Also, the three human ALD isoenzymes (ALD-A, ALD-B and ALD-C), which are differentially expressed in the tissues, have been observed to have aberrant expression in several diseases and types of cancer [115]. ALD-B mRNA levels may also provide additional information on disease prognosis in patients with hepatocellular carcinoma [116].

Notably, these glycolytic isoenzymes have different kinetic properties and are differently regulated [107,117–119]. This likely allows a modulation of glycolytic metabolism in response to various physiological and environmental signals. A notable feature of these isoforms is their close association with several diseases, even with pathological pregnancy [109,120–127]. Also, they can exhibit both enzymatic and non-enzymatic functions [128] and their expression is predominant in embryonic/foetal and proliferating tissues [129–131]. However, during development, they are gradually supplanted by other isoforms in specific tissues [132]. Tissues such as embryonic ones have a metabolic phenotype characterized by dependence on aerobic glycolysis, which allows them to support rapid cellular proliferation and metabolic demand, a feature reminiscent of what is seen in cancer [125,132,133], as we will describe in the next sections. In non-cancerous cells, such as immune cells, aerobic glycolysis is also observed under specific conditions, especially where rapid proliferation is necessary [134].

Although aerobic glycolysis is relevant in the first stages of embryonic development, as this process progresses, a rewiring of the cellular metabolic state occurs. This involves a metabolic switch between glycolysis and mitochondrial OXPHOS [133]. Contrary to this, in tumour cells, there is a switch from glycolysis and oxidative respiration to aerobic glycolysis, as well as restoring the expression of some embryonic isoforms of glycolytic enzymes [135,136].

#### 2.2.1. Regulation activity of enzymes

In glycolysis, allosteric activity regulation occurs mainly at three enzymes of the pathway, HK, PFK and PK [137,138] (figure 1). These enzymes are either activated or inhibited by molecules (ligands) such as Pi, ATP, ADP and AMP, glycolytic intermediates and other metabolites (mainly of energy and carbon metabolism). Each of these enzymes serves as a finely regulated and critical site; adjusted in response to environmental changes both inside and outside the cell [25,139].

##### 2.2.1.1. Hexokinase

HK is the first enzyme of glycolysis, catalysing the phosphorylation of glucose by ATP to glucose-6-phosphate (G-6-P) [140]. This HK-catalysed reaction not only is important for activating glucose necessary for its catabolism in the pathway, but also may restrict the glucose level in cells [27]. HK is usually feedback-inhibited by its product G-6-P, that binds at the C-terminal domain (catalytic domain) so occupying the putative binding site for ATP, whereas the 6-phosphoryl group may overlap with the binding site for Pi at the N-terminal domain (regulatory domain) [141] (figure 1). An elevated level of G-6-P acts as an indication that the cell does not need to obtain glucose as an energy source or as a source of biosynthetic precursors [142]. Additionally, the HK activity is also regulated when the PFK function is suppressed. When PFK is inactive, the concentration of fructose-6-phosphate (F-6-P) increases, which in turn leads to increasing G-6-P levels, due to the existing balance of it with F-6-P, an equilibrium catalysed by PGI [142]. Importantly, other molecules such as orthophosphate (Pi) can influence HK activity [141]. The binding of Pi to the N-terminus of HK suppresses the inhibition caused by G-6-P, by directly competing with G-6-P at the N-terminal domain, resulting in increased HK activity which may contribute to an increased glycolytic flux [141,143]. The modulation of HK’s activity, as well as its spatio-temporal location, by reactive oxygen and nitrogen species (ROS and RNS) has been documented in humans, and in other organisms such as certain parasites, kinetoplastids and apicomplexans, of clinical significance. This redox regulation of HK is considered a potential for the treatment of some diseases, especially as part of therapeutic approaches with low toxicity [144].

##### 2.2.1.2. Phosphofructokinase

PFK catalyses the conversion of F-6-P and ATP to fructose-1,6-bisphosphate (F-1,6-BP) and ADP. This enzyme is considered a key regulator of glycolysis [145], because its activity is modulated by a multitude of allosteric effectors that integrate glycolytic flux in the network of carbon metabolism and cellular bioenergetics [146,147] (figure 1). It is allosterically inhibited by high levels of *ATP*, leading to a reduction in its affinity for its substrate F-6-P. However, AMP reverses the inhibitory action of ATP, so that the enzyme’s activity increases when the ATP/AMP ratio is reduced. This would imply that glycolysis is stimulated as the energy load falls [142]. Indeed, inhibition of PFK by ATP is part of a negative feedback loop that limits the glycolytic flux under aerobic conditions (Pasteur effect). PFK is also regulated by *fructose-2,6-bisphosphate (F-2,6-BP)*, a potent allosteric activator of the enzyme. F-2,6-BP is produced (and hydrolysed) by different isoforms of the bifunctional enzyme 6-phosphofructo-2-kinase/fructose-2,6-bisphosphatase (also known as PFK-2, to distinguish it from the glycolytic enzyme PFK, also known as PFK-1). Unlike ATP, the binding of F-2,6-BP increases the enzyme’s affinity for F-6-P and enhances the glycolytic flux [148]. In muscle, other factors inhibit the activity of PFK, such as lowering the *pH*, which increases the protonated state of the enzyme and consequently augments the binding of ATP and thus its inhibitory effect. H^+^-induced inhibition of this enzyme prevents excessive lactic acid formation and a subsequent abrupt drop in blood pH (acidosis) and muscle damage from lactic acid build-up [149]. The inhibition during the decrease in pH serves as a mechanism of coordinated control of the glycolytic flux during altered work states [149].

Other molecules, such as *citrate*, the first intermediate in the TCA cycle after input of glycolytically derived acetyl-CoA, have a modulating effect on the catalysis by PFK [146,150]. In eukaryotes, the amino acid residues that make up the allosteric binding site for citrate are located in both the N- and C-terminal regions of the enzyme [150] (figure 1). In addition, the synergistic action of citrate with ATP is apparently mediated by depolymerization of the active tetrameric enzyme into inactive dimers [149]. Physiological significance is given in that elevated citrate levels could mean that biosynthetic precursors are abundant, and no additional glucose should be catabolized for this purpose.

The ability of *fatty acids* to suppress the glycolytic flux through PFK has also been described [146]. Through some studies, it was shown that acyl-CoA, a branching point metabolite in the anabolic and catabolic metabolism of fatty acids, controls the catalytic rate of PFK. Long-chain fatty acyl-CoAs (LCFA) promote the inhibition of PFK (figure 1). However, palmitoyl-CoA-mediated acylation of PFK and inhibition of its catalytic activity were reversible in the presence of acyl-protein thioesterase-1 (APT-1) [146], an enzyme that cleaves off lipid modifications on proteins [151], which suggests that this regulatory mechanism is likely to respond efficiently to rapid metabolic transitions [146]. Importantly, the presence of palmitoyl-CoA promotes the association of PFK both with membrane vesicles and with some of its regulatory interacting proteins, such as calmodulin. Fatty acyl-CoAs promote alterations in PFK activity that is indicated either through its interaction with the membrane and/or through association with regulatory proteins. This regulation would allow the integration of glycolysis with lipid metabolism and could thus be relevant to maintain metabolic balance [146], in health conditions and especially also in those disease conditions where there is accumulation of fatty acyl-CoAs [152–154].

##### 2.2.1.3. Pyruvate kinase

PK is the enzyme involved in the last step of glycolysis, catalysing the transfer of a phosphate group from phosphoenolpyruvate (PEP) to ADP, generating a pyruvate molecule and an ATP molecule [118]. Mammals possess four PK isoforms: PK of red blood cells (PKR), PK of the liver (PKL), PK of muscle and brain (PKM1) and PK of early foetal tissue and adult tissues (PKM2), each with unique expression and activity-regulation patterns. The PKM2 isoform is responsible for supporting anabolic metabolism and most adult tissues express this isoform, in both health and disease [118]. Additionally, being closely linked to tissue repair and regeneration, as well as cancer and inflammation, the role of PKM2 has received considerable attention [130,155,156]. Like some other glycolytic enzymes, the enzymatic activity of PKM2 is allosterically regulated by both intracellular signalling pathways and metabolites. This implies that PKM2 integrates metabolic and other signals to modulate glucose metabolism according to the needs of the cell [118,157].

Structurally, mammalian PK is a homotetrameric protein of identical subunits, arranged in a dimer–dimer configuration [157]. Each monomer, in turn, contains an active site and is made up of three main domains (A, B and C), plus a small N-terminal domain. Domains A and B form the active site, where the Mg^2+^–ADP substrate complex binds, while domain C is involved in forming the dimer–dimer interface of the fully associated tetramer [118,157]. Although PK is more active as a tetramer, the PKM2 isoform is not a constitutive tetramer; its tetrameric conformation is subject to reversible dissociation and inactivation dependent on the presence and binding of effector molecules, such as ATP and F-1,6-BP, amino acids and other non-glycolytic metabolites [118,158].

*F-1,6-BP* is considered the major allosteric regulator/activator of PK (figure 1). Its binding induces an increase in PK’s affinity for PEP, promotes tetramerization and stabilizes the tetrameric enzyme in the active state. This molecule binds to the allosteric effector binding site located in the C domain [159], inducing a conformational change that involves the rotation of the B and C domains of each subunit and a rotation of each subunit in the tetramer. These conformational changes within subunits greatly not only influence the interactions between neighbouring subunits, but also enhance their stability and rigidity [157]. Notably, almost all PK isoforms expressed in humans, except PKM1, contain per subunit one binding site that is specific for F-1,6-BP. PKM1 does not bind F-1,6-BP due to structural differences (it has a glutamate at position 433 instead of lysine) in the F-1,6-BP-binding pocket [160,161].

Other molecules such as *amino acids* can also act as PKM2-specific allosteric inhibitors/activators, using the same allosteric site [157,162] (figure 1). Amino acids such as phenylalanine (Phe), alanine (Ala), tryptophan (Trp), methionine (Met), valine (Val) and proline (Pro) act as inhibitors, while histidine (His) and serine (Ser) act as activators [162]. In the case of amino acids that act as inhibitors, Ala, Phe and Trp, X-ray structural studies of PKM2s complexed with them showed that their binding reduces the enzyme’s activity by decreasing its affinity for PEP as a result from stabilizing the tetramer in an inactive state (T-state) where the active site cleft is held in an open conformation [162]. Additionally, Ala, originating from the transamination of pyruvate, could also inhibit PK by preventing PEP from being converted into pyruvate [163]. Other amino acids such as l-cysteine also promote the tetrameric dissociation of PKM2 into dimers/monomers in cells, resulting in impaired activity of PKM2 [164].

On the other hand, the activating amino acids, Ser and His, stabilize the PKM2 tetramer in its active conformation (R-state) [162,165]. The difference in exerting an inhibitory or stimulatory action is mainly due to the binding of the amino acids in the allosteric pocket triggering rigid-body rotations that stabilize either the T or the R state [162]. The binding of these inhibitory and stimulatory amino acids could be related to a regulatory mechanism that allows PKM2 to finely adjust to physiological conditions associated with the concentrations of these amino acids. In this sense, this would imply that this allostatic regulation of PKM2 could act as an important amino acid sensor in metabolic reprogramming and influencing cell fate. Since PKM2 is at the intersection of several metabolic pathways, it could influence the feeding of some metabolic pathways (such as the TCA cycle) in response to amino acid availability [162]. Additionally, under conditions/in the presence of growth factors where its inactive dimeric state is induced, PKM2 can even enter the nucleus and promote transactivation of HIF-1 target genes, many of which code for metabolic enzymes [166,167]. Other studies have shown that isoform PKM1 is also allosterically inhibited by some amino acids, such as Phe and its main metabolite, phenylpyruvate [168,169]. Decreased brain PK activity may be related to the reduction in glucose metabolism observed in the brain of phenylketonuric patients and could be one of the causes of the neurological dysfunction observed in these patients [168].

*Non-glycolytic metabolites* such as succinyl-5-aminoimidazole-4-carboxamide-1-ribose-5′-phosphate (SAICAR), an intermediate of the de novo purine nucleotide synthesis (NPNS) pathway, have a stimulatory effect on PKM2 activity and promote cancer cell survival in glucose-limited conditions [170,171] (figure 1). SAICAR promoted activation and increased the catalytic turnover number (*k*_cat_) of PKM2 by twofold to threefold and reduced the *K*_m_ for PEP up to 10 times [170]. Importantly, over 100 kinases are phosphorylated and activated by PKM2-SAICAR, including Erk1/2, which in turn phosphorylates PKM2 and sensitizes it to SAICAR binding. SAICAR regulation could be a mechanism by which tumour cells not only coordinate different metabolic pathways under limited nutritional conditions but also couple cell proliferation with intracellular metabolic status [171]. This allosteric regulation of PKM2 could allow cells to adjust energy generation in response to nutritional demands. Under conditions rich in glucose, the diversion of glycolytic intermediates towards biosynthetic processes, such as the PPP, would contribute to cell growth. However, under conditions of nutritional stress, the diversion of glycolytic intermediates towards biosynthetic processes could lead to cellular energy depletion below the required level [170,171]. The SAICAR–PKM2 interaction could be a molecular mechanism to maintain a fine balance of metabolism since connecting the activity of PKM2 with an intermediate of the NPNS pathway could provide cells with precise control of their metabolism in demanding conditions [170].

Like on many other enzymes, *post-translational modification* is used to exert a regulatory effect on PK (figure 1). In the liver, the possible hormonal control of PK activity and gluconeogenesis has been documented. In hepatocytes, glucagon and epinephrine activate protein kinase A (PKA), which serves as a covalent modifier by phosphorylating PKM2 that results in inhibition of the enzyme’s activity and stimulation of gluconeogenesis [172,173]. In contrast, insulin activates phosphoprotein phosphatase I and subsequently causes dephosphorylation and activation of PKM2 and increased glycolysis. This modification of PK is accompanied with the opposite effect on gluconeogenesis enzymes. This supports the idea that this regulatory system is responsible for preventing a futile cycle by averting the simultaneous activation of PK and other enzymes involved in these opposing pathways [172,173]. It should be noted that in certain pathologies, such as cancer, PK may undergo other post-translational modifications such as deacetylation, acetylation and succinylation, which can affect its activity [174]. However, this will be covered in more depth in the following sections.

PK is also allosterically inhibited by *ATP*; however, this allosteric inhibition and the N-terminal phosphorylation by ATP is unique to PKL and PKR [175] (figure 1). This inhibition represents both feedback inhibition by an end product of the glycolytic pathway, as well as the susceptibility of the energy-producing catalytic reactions to the intracellular energy state [175]. During gluconeogenesis, ATP levels are elevated in the cell and exert an inhibition on PK activity. This in turn allows oxaloacetate to be converted to PEP rather than being diverted to the production of pyruvate, which would create a futile cycle [163]. Other molecules as such *acetyl-CoA* also inhibit PKL and PKM1, preventing the recycling of PEP by these enzymes, and consequently exert control on the rate and direction of hepatic carbohydrate metabolism [176].

## 3. Glycolytic pathway in various organisms

Generally, glycolysis takes place in the cytosol of prokaryotic and eukaryotic cells [72]. However, as we will see in the following paragraphs, the partial location of glycolytic enzymes in other subcellular compartments has been reported for some eukaryotes, mostly parasites [72,101,177–183]. In bacteria, glycolysis represents one of several pathways by which bacteria can catabolize glucose, and it is the pathway that is most associated with anaerobic or fermentative metabolism in these organisms [184]. However, several of the glycolytic enzymes have dual locations, including the cell’s surface where the proteins may not participate in glycolysis but act as virulence factors [185,186]. For example, in some pathogenic bacteria, surface-exposed enzymes such as GAPDH, PGM, PK and lactate dehydrogenase (LDH) can bind the host’s plasmin/plasminogen [185,186]. In the case of GAPDH expressed on the surface of other bacteria, it can bind other host proteins such as lactoferrin, transferrin, laminin, fibronectin, among others [187].

Modified versions of this glycolytic or EMP pathway are present in anaerobic archaea and cyanobacteria [97,188]. Even some hyperthermophilic organisms use up to three different pathways for glucose metabolism, including some variants of the EMP and ED pathways [97,189–191]. The presence of these variants of the EMP pathway is associated with an important role in adaptation to environmental conditions [190,192]. Although the classic EMP pathway and its modified versions in archaea differ markedly in some enzymes, the process in these organisms proceeds basically through the same known intermediates as in the classic EMP pathway in bacteria and eukarya, highlighting an ancestral character [97]. However, the modifications are mainly found in the upper part of the EMP path and the high conservation of the second half of the pathway could thus represent its nature as the oldest part of the pathway, which could be related to a primordial origin of gluconeogenesis.

In eukaryotes, exceptions are intracellular parasitic Enterocytozoonidae microsporidia, which lack basic pathways necessary for carbon metabolism, including glycolysis, and obtain energy directly from their host, during the dispersive spore stage. The loss of the glycolytic pathway is the result of an adaptation to the lifestyle of intranuclear parasitism [193,194]. Nonetheless, the retainment of one or several copies of the genes encoding a HK enzyme is a common characteristic in the Enterocytozoonidae parasites. Copies of HK proteins could be secreted by these intranuclear life parasites to modify host metabolism and increase metabolic supply for the pathogen [194,195].

Partial location of glycolytic enzymes in other subcellular compartments than only cytosol has been reported for other pathogens [72,101,177–183]. Compartmentalization of a major part of the glycolytic pathway in peroxisome-related microbodies known as glycosomes has been documented for kinetoplastid and diplonemid protists, some of them causing diseases in humans [101,180,196,197]. This compartmentalization is associated with the absence of most commonly known regulatory mechanisms of some glycolytic enzymes, such as HK and PFK [198,199]. Likewise, flagellar PGK-like and GAPDH-like proteins have also been identified in kinetoplastids such as *Trypanosoma brucei* and *Bodo saltans* [101,196]. Although the significance of these glycolytic enzymes in the flagellum is unknown, it has been suggested that this localization may be related to a change in glycolysis regulation and compartmentalization during kinetoplastid evolution. An alternative proposal is that these proteins can act as ‘functionally flexible’ enzymes, performing various cellular tasks unrelated to glycolysis, but adapted to the environmental and nutritional requirements of different kinetoplastid groups, within which they are also conserved [101]. In kinetoplastids such as *Leishmania donovani* and *T. brucei*, an HK is not only expressed in glycosomes, but also in or at the flagellum [200,201]. In *Leishmania*, the HK in its flagellar pocket functions as a haemoglobin (Hb) and plasminogen receptor which seems to be key during haemoglobin endocytosis [200], whereas in *T. brucei* the function of this HK in the basal body of the flagellum is still unknown [201]. However, it has been proposed that this flagellar HK could be involved in functions such as glucose phosphorylation, Hb binding or sugar sensor, which may or may not be interconnected [202]. Also, some ENO enzyme has been detected on the external side of the plasma membrane of *Leishmania mexicana*, so it has been suggested to have similar functions as in some other prokaryotic and eukaryotic cells [203]. In helminthic parasites such as *Schistosoma*, glycolytic enzymes present on the external surface of the parasite are supposed to function in association with immune modulation and blood clot dissolution that promote parasite survival [204], while in other parasites such as the apicomplexan *Toxoplasma gondii*, part of the ENO is directed to the nucleus of actively replicating parasites where it acts as a transcriptional regulator of genes related to host metabolism, cell invasion and modulators of immune responses [205].

On the other hand, in various stramenopile organisms, such as *Blastocystis* and *Phytophthora infestans*, some enzymes involved in the last stage of glycolysis are present in the mitochondrial matrix [72,206]. In these organisms, the second half of the glycolytic pathway, responsible for the metabolism of the C3 part produced from glucose and the ‘pay-off’ process, is targeted to mitochondria. Apparently, this feature is unique to stramenopiles and could be a synapomorphy for this group of eukaryotes [72]. In phytopathogenic ascomycota and basidiomycota fungi, some glycolytic enzymes have a dual localization, in cytosol and peroxisomes [100,207]. In the fungus *Ustilago maydis*, GAPDH and PGK are partially targeting to peroxisomes and they are important for virulence and growth [100]. Similarly, proteomics of *Neurospora crassa* glyoxysomes, which are specialized peroxisomes, identified GAPDH as a ‘peroxisome-targeting signal (PTS)-less’ peroxisomal protein [207]. In pathogenic yeasts such as *Candida glabrata*, GAPDH and ENO have been found on the surface of the cell wall, possibly fulfilling functions related to cell wall maintenance and the pathogenesis of these fungi [208].

Pathogenic virus infections have been found to induce a drastic alteration of the cellular metabolism of the host. Some viruses hijack and promote a metabolic reprogramming of the infected cell, through activation of some specific metabolic pathways for their replication and spread [209–211]. Most viruses that alter cellular glucose metabolism induce aerobic glycolysis, also known as the Warburg effect, associated with an increase in virus replication, since it not only provides energy and specific cellular substrates for viral particles and creates niches for viral replication, but also increases the survival of infected cells [209,210]. Unlike tumour cells, where numerous mutations and disturbance of the glycolytic flux are observed, cells infected by adenovirus and some other viruses upregulate only key metabolic nodes to replicate effectively. These metabolic alterations can vary within the same family of viruses or even depend on the type of host cell that is infected [210].

In humans, glycolysis occurs mainly in the cytosol of the cell; however, several reports document the localization of some glycolytic enzymes in various subcellular locations such as the nucleus [121,212–214], mitochondria [215,216], at the cell surface [217,218], associated with the plasma membrane as a glycolytic metabolon [219] and in other locations in the cell [220]. Alterations in the localization and expression are generally associated with the development and progression of some diseases [221–227], and used as common markers of some of these conditions [228,229]. In the present work, many aspects related to the role of glycolytic enzymes in the development of some diseases of great clinical relevance, such as cancer, neurodegenerative and parasitic, will be addressed, emphasizing their possible usefulness as possible tools for diagnosis, prognosis and development of therapeutic strategies.

## 4. Glycolytic enzymes in the development of pathologies

### 4.1. Neurodegenerative diseases

Neurodegenerative diseases (NDDs) are a heterogeneous group of conditions that result in the progressive loss of the structure and function of neurons through a process of neurodegeneration. This process ultimately leads to motor impairment, cognitive disabilities and dementia [230]. These diseases affect millions of people around the world, representing the equivalent of 12% of all deaths in the world [231]. The World Health Organization (WHO) estimates that by 2040, some NDDs, such as Alzheimer’s and Parkinson’s, will overtake cancer to become the second leading cause of death after cardiovascular diseases [232]. In the United States alone, the costs of these NDDs, which are attributed mainly to medical expenses and economic losses, exceed US$655 billion annually [233,234], meaning a great impact economy. Currently, the lack of cures, along with the limited availability of effective treatments, are factors that contribute significantly to these costs [233].

These diseases are considered incurable because there is no way to reverse neurodegeneration. Some factors such as oxidative stress and inflammation are main causes of these diseases [235]. Additionally, some processes such as the assembly of atypical proteins (proteinopathy), cell death and changes in metabolism are common in many neurodegenerative disorders [14,230]. Alzheimer’s disease (AD) patients are characterized by having multiple abnormalities in their metabolism, including that of glucose [236–238], and are probably predisposed to NDDs with age [236].

Glycolysis is related to important brain processes. Its contribution through rapid ATP generation, the synthesis of biosynthesis precursors and cellular homeostasis, attributes relevant influences such as action potential conduction and synaptic transmission, as well as postsynaptic activity and redox homeostasis [26]. However, the role of glycolysis can vary depending on the type of nerve cell. Cells use this pathway in a complementary manner to meet both their metabolic demands and function in the brain [239–242]. In neurons, which rely primarily on OXPHOS, the glycolytic flux is low, which is crucial to maintain the redox balance in the cell [239]. In other cells such as astrocytes, commonly described as glycolytic cells, glucose is intensively used and converted to lactate via aerobic glycolysis, even in the presence of sufficient oxygen. This lactate is exported to the extracellular space and subsequently shuttled to neurons where it is used as fuel for OXPHOS and to help maintaining the redox balance [241]. For their part, microglia adapt their metabolism depending on their activation state. In resting state, they maintain a balance between glycolysis and OXPHOS. However, when activated in situations such as inflammation, they adopt a predominantly glycolytic metabolism, which allows them to support their energetic and functional demands [242].

Glycolytic pathway alterations in cells of the nervous system and peripheral cells have been observed in the early and advanced stages of some neurodegenerative diseases [14,26,243,244], so it has been suggested that they may have a critical contributing role to the pathogenesis of these diseases, and in turn could serve as a tool for diagnosis and design of therapeutic strategies for these pathologies [26,245,246].

#### 4.1.1. Alzheimer’s disease

AD is one of the most common causes of dementia and is considered an important public health problem with great implications for individuals and society [247]. According to Alzheimer’s Disease International (ADI), 75% of individuals with dementia remain undiagnosed, with rates potentially reaching up to 90% in low- and middle-income countries (LMICs). Likewise, Dementia Forecasting Collaborators and ADI estimated the prevalence of dementia at around 57 million people worldwide [248,249], which is expected to triple by 2050. Also, it is anticipated that the proportion of people living with dementia in LMICs is expected to reach 71% by 2050 [250].

**Figure 2.**
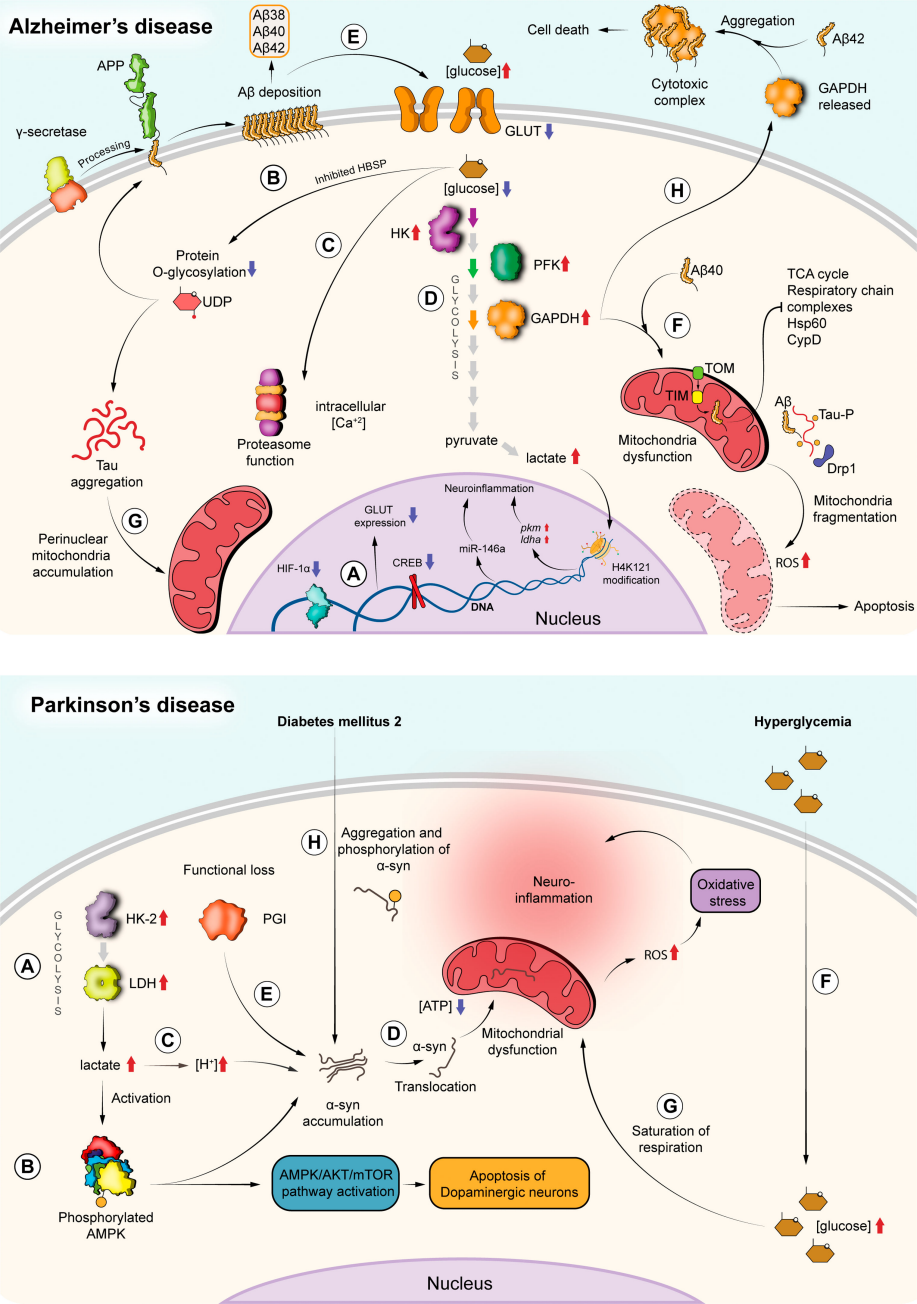
Molecular processes in neurodegenerative diseases. *Alzheimer’s disease (AD)*. (*a*) Expression of the glucose transporters GLUT1 and GLUT3 is compromised in neurons and astrocytes due to downregulation of the transcription factors HIF-1α and CREB. (*b*) The lower concentration of intracellular d-glucose affects the HSBP pathway, resulting in decreased formation of UDP-GlcNAc, and subsequently reduced *O*-glycosylation of Tau and APP proteins, which leads to their aggregation and deposition. (*c*) Additionally, an activation of the inflammatory response and an increase of the Ca^2+^ concentration are also consequences of HSBP inhibition. (*d*) The reduction of glucose transport allows the upregulation of glycolytic enzymes. (*e*) A positive feedback is possible since Aβ depositions affect GLUT expression, allowing more deposition of Aβ plaques. (*f*) Aβ plaques also affect mitochondrial functionality. Aβ42 passes through the TIM/TOM transporter and inhibits TCA-cycle enzymes and respiratory complexes. Furthermore, phosphorylation of Tau by Aβ42 in the presence of Drp1 leads to mitochondrial fragmentation, the release of ROS and apoptosis. (*g*) Tau accumulations are also responsible for the perinuclear localization of mitochondria and their absence from the axon. (*h*) GAPDH is involved in the progression of AD since it is released extracellularly to form complexes with Aβ42 that lead to cytotoxicity and cell death. Intracellularly, this enzyme also interacts with Aβ40 to disrupt mitochondria and finally induces death of the cell. *Parkinson’s disease (PD)*. (*a*) Upregulation of some glycolytic enzymes, such as HK-2 and LDH-A that induce the overproduction and accumulation of lactate. (*b*) The activation of AMPK by phosphorylation occurs because of lactate accumulation. This event regulates negatively the mTOR pathway and subsequently induces apoptosis of dopaminergic neurons. Phosphorylated AMPK also contributes to α-syn accumulation. (*c*) The excess of lactate promotes acidification. (*d*) As a consequence, α-syn accumulates in the cytosol and migrates to mitochondria where it promotes dysfunction and reduced ATP production. (*e*) PGI also contributes to α-syn aggregation and neurodegeneration. (*f*) Hyperglycaemia causes neuroinflammation and (*g*) saturation of mitochondrial respiration. These changes lead to ROS production and oxidative stress. (*h*) Another consequence of hyperglycaemia (in this case in type 2 diabetes mellitus) is the accumulation and phosphorylation of α-syn in pancreatic and neuronal cells.

In AD, glucose hypometabolism is considered an invariant biomarker and is related to the severity of this disease [245]. Although the exact cause of this metabolic condition is unknown, it has been assumed that it originates from a decreased glucose uptake due to loss of GLUT transporter expression [251,252] and dysfunction of mitochondria in the brain [253,254]. In some brain regions such as the hippocampus and temporal cortex, involved in memory, hypometabolism of glucose is observed during the initial stage of AD [255], while other areas such as the parietal-temporal lobe, posterior cingulate cortex and the frontal areas, related to the senses and emotions, show a significant decrease in glucose metabolism during the progression of the disease [256–258]. As we will describe in the following sections, the glycolytic pathway is involved in different aspects linked to the development and progression of AD (figure 2).

##### 4.1.1.1. Amyloid beta formation and Tau interaction

Studies involving brain tissue obtained at autopsy from patients with AD have revealed decreased expression of neuronal (GLUT3) and astrocytic (GLUT1) glucose transporters [251,252] and their lower expression could be attributed to HIF-1 and CRE-binding protein (CREB) downregulation [259,260] (figure 2). The loss of these transporters would not only cause ATP production to decline by 50%, with a tendency to decrease with disease progression [261], but also promote the accumulation of amyloid plaques, composed of the amyloid precursor protein (APP) product amyloid beta (Aβ), and the intracellular aggregation of the Tau protein [262,263], both distinctive clinical manifestations of this disease [264]. The decrease in the d-glucose concentration in neurons could have an impact on neuroprotective signalling pathways such as the hexosamine biosynthetic pathway (HBSP) [265]. This pathway is involved in the synthesis of uridine 5′-diphosphate-*N*-acetylglucosamine (UDP-GlcNAc), a molecule used for *O*-GlcNAc post-translational modification of a wide range of proteins, including Tau and APP [266]. The *O*-GlcNAcylation of these proteins functions as a neuroprotective mechanism since it decreases Tau hyperphosphorylation, which is responsible for the formation of neurotoxic Tau oligomers, and decreases the formation of Aβ due to inactivation of γ-secretase and/or degradation of APP [267,268]. In the absence of glucose, inhibition of the HBSP would occur, which leads to a decrease in the *O*-glycosylation of Tau and APP, and the subsequent aggregation of Tau and deposition of Aβ [265], as well as activation of an inflammatory response and inhibition of other cellular processes (such as proteasome function and alteration of intracellular Ca^2+^ levels) [269] (figure 2). Additionally, the change in expression of GLUT transporters could induce an increase in the expression and activity of some enzymes such as PK, ALD and GAPDH in neurons [270]. However, this could be a cellular strategy to cope with the reduced glucose availability caused by a reduction in the GLUT1/3 transporters [14] (figure 2).

Glucose hypometabolism, caused by the decrease in GLUT transporters, would not only occur in neuronal cells and astrocytes, but also in other important cell populations of the brain in which they are expressed, such as endothelial cells of the blood–brain barrier (BBB) and oligodendroglial cells, and are involved in the maintenance of energy supply to neurons [265,271]. The arrest of glucose metabolism and consequently processes dependent on ATP production can occur in these cells, leading to reduced cerebral blood flow (hypoperfusion), altered homeostasis of the brain microenvironment, and subsequent synaptic dysfunction, neurodegeneration and cognitive deficits [272].

Importantly, the low GLUT expression may be linked to Aβ formation [273–275]. Some studies suggested that Aβ plaques lead to the degeneration of capillaries, which may in turn affect GLUT expression [274]. Alternatively, there could be a mechanistic link between β deposition and inhibition of glucose transport. This is because Aβ deposition induces oxidative damage, through lipid peroxidation, in the plasma membrane. The generation and conjugation of some peroxidation products, such as 4-hydroxynonenal, to GLUT3 would prevent glucose transport and subsequent ATP depletion [273]. This could lead us to think that a kind of negative feedback loop occurs, in which the decrease in GLUT expression would promote the deposition of Aβ plaques; in turn, this deposition could promote capillary degeneration and modulate GLUT expression and glucose uptake and inhibition of glucose-dependent pathways.

It should be noted that the formation of Aβ alters the function of the mitochondria, promoting their dysfunction and increased ROS production, another distinguishing feature of AD [254,276–279] (figure 2). In neurons, Aβ is generated by sequential cleavages of its larger amyloid-beta precursor protein (AβPP), which can give rise to different Aβ species (Aβ38, Aβ40 and Aβ42), which differ in their lipophilic properties and tendencies to form Aβ oligomers and aggregates [277]. Although the Aβ40/Aβ42 ratio is extracellular and is the one with the greatest clinical relevance in AD [280], other Aβ species misfold and self-aggregate into oligomers of various sizes and can be disrupted within various subcellular locations such as the mitochondria [281,282]. Some species such as Aβ42 can enter mitochondria facilitated by TIM/TOM transporters. Inside, Aβ can inhibit the function of enzymes that participate in the TCA cycle, respiratory chain complexes (I, II and IV), and the matrix protein HSP60 chaperonin [281,282]. Additionally, it can inhibit the function of cyclophilin D (CypD), i.e. mitochondrial peptidyl-prolyl cis-trans isomerase, which regulates the mitochondrial permeability transition pore (PTP) and mitochondrial energy coupling [276–278,283]. Also, the interaction of Aβ with the dynamin-related protein 1 (Drp1), a protein involved in the fission of mitochondria, contributes to excessive mitochondrial fragmentation, abnormal mitochondrial dynamics and synaptic damage [284,285]. Alternatively, Aβ also induces excessive mitochondrial fission by stimulating the interaction of Drp1 with mitochondrial fission protein 1 (Fis1), a mitochondrial outer membrane protein that is involved in mitochondrial fission [286]. All these Aβ-mediated mitochondrial disruption events and increased ROS production [282] eventually lead to apoptosis, axon withdrawal, synaptic dysfunction, denervation, damage and cognitive decline, all characteristic processes of AD [278].

A study in which human skin fibroblast cell strains from patients with Alzheimer’s were used has shown that the activity of the glycolytic enzyme GAPDH was decreased by 27% as compared with age-matched controls, in both nuclear and post-nuclear fractions [287]. Although the causes of this loss of GAPDH activity are unknown, it has been suggested that it may be a consequence of the direct interaction of this enzyme with proteins related to AD, such as Aβ and AβPP. This interaction renders the enzyme catalytically less active [288,289] resulting in a deficiency in energy production and subsequent development of AD [287]. Also, GAPDH expression levels, as well as organelle distribution and thermal stability were affected by αβPP [290].

The decrease in GAPDH activity in nuclear fractions [287] may be related to non-glycolytic (moonlighting) functions attributed to GAPDH, which have relevant roles in various diseases, including NDDs. In the nucleus, non-glycolytic functions of GAPDH are linked to the regulation of some processes such as cell death, autophagy, DNA repair and protection of telomeres [222,291].

The hypothesis has been proposed that the binding of GAPDH to some proteins associated with NDDs can stimulate its translocation to the nucleus, causing it to induce apoptosis and cell death, a key event in AD disease [222,292]. All of this implies that mammalian cells contain a mechanism by which GAPDH catalysis is inhibited so that it acquires a function different from its enzymatic activity, triggering apoptosis [222,287]. Although the exact mechanisms regulating nuclear GAPDH activity remain unclear, it has been postulated that the presence of a specific nuclear metabolite, as well as post-translational modifications and fluctuations in GAPDH interaction states with other proteins, could regulate the activity of this enzyme [293].

Glycolysis has also be linked with aggregation and/or phosphorylation of the microtubule-associated protein Tau, the other hallmark protein of AD [263,294–296]. Studies focused on aerobic glycolysis (AG), i.e. the catabolism of glucose without OXPHOS, and Tau pathology have revealed that loss in AG, related to aging, induces a decrease in synaptic plasticity and neuroprotection, facilitating the aggregation of Tau in individuals with amyloid burden. This may occur because the decrease in AG implies a direct loss of its biosynthetic and neuroprotective functions, associated among many other things with reducing oxidative stress [297]. Although the exact link in this relationship is unknown, it is believed that Tau deposition alters some processes that require AG such as synaptic plasticity. Alternatively, loss of AG would accelerate tauopathy [263]. In other reports based on cells and a tauopathy mouse model, it was demonstrated that the loss of AG promotes an increase in Tau phosphorylation mediated by the activation of the p38 mitogen‐activated protein kinase (MAPK) pathway via the participation of the ASK1 kinase [294,298], ultimately causing neuronal apoptosis mediated by caspases 12 and 3 [298]. This is related to the fact that p38 is closely linked to the cellular stress response and inflammation, its activation normally causing cell cycle arrest or apoptosis as a common response [299]. All of this suggests that glucose deprivation, by acting as a metabolic stress factor, can influence Tau metabolism and, consequently, be a key and pleiotropic active regulator in AD and related neuropathologies [294].

Paradoxically, it has also been documented that high glucose induces Tau hyperphosphorylation, through activation of the mTOR pathway and regulation of N^6^-methyladenosine (m6A) mRNA methylation [295,300]. Under hyperglycaemic conditions, caveolin-1 (Cav-1)-mTOR/S6K signalling is involved in the formation of Tau hyperphosphorylation. Apparently, high glucose levels inhibit the expression of Cav-1 and increase the activation of the mTOR/S6K signalling pathway and subsequently the phosphorylation of Tau [295]. On the other hand, alteration in glucose capture can regulate the m6A modification of diacylglycerol kinase eta (DGKH) mRNA through AlkB Homolog 5, RNA Demethylase (ALKBH5), resulting in reductions in both DGKH mRNA and protein levels. When the expression of DGKH is inhibited, protein kinase C-α (PKC-α) is activated and promotes Tau phosphorylation and ensuing diabetic cognitive dysfunction [300]. All of this supports the idea that glucose metabolism has a dichotomous role in Tau metabolism/phosphorylation. Also, that the inhibition of pathways activated by the alteration of glycolysis, such as p38/ASK, Cav-1/mTOR/S6K or ALKBH5, could be taken into consideration as therapeutic targets to stop cell death in response to energy metabolic stress, as well as inhibition of neurofibrillary tangles of Tau. In other studies based on the arrayed CRISPR method, where genes related to Tau aggregation were studied, an association of some *pklr* and *pgk1* genes with Tau aggregation was found [301]; however, the nature of this association is still unknown.

Similar to Aβ, Tau has been associated with mitochondrial dysfunction in AD [302,303]. Tau aggregation leads to the alteration of mitochondrial transport within the neuron and bioenergetics, contributing to axonal degeneration. In bioenergetic alteration, it induces interference with the function of respiratory chain enzymes and the TCA cycle, similar to how Aβ does [303]. However, Tau also affects the mitochondrial distribution, by contributing to an increase in their retrograde transport, leading to an accumulation of perinuclear mitochondria and a decrease in their number in the axon [302]. Beyond the mitochondria, oxidative stress activates signalling pathways involved in APP or Tau processing. Oxidative stress increases the expression of β-secretase, through activation of c-Jun kinase and mitogen-activated protein kinase (MAPK), and increases Tau phosphorylation by activation of glycogen synthase kinase 3-β (GSK3-β) [304], potentiating the inhibition of glucose transport by Aβ plates, leading to a positive feedback loop.

##### 4.1.1.2. Resistance to amyloid beta-dependent apoptosis

The upregulation of glycolysis has been observed in the early phase of AD. During this phase, neuronal cells ‘prefer’ to burn glucose by glycolysis, like tumour cells do [305]. Although the exact reason is unknown, it is thought as an extreme attempt of opposing imminent death. In some nerve cell lines that exhibit resistance to Aβ-dependent apoptosis and accumulation, an increase was observed in the activities of HK, PFK and isoform A of LDH (LDH-A) and the expression of the glucose transporter GLUT and pyruvate dehydrogenase kinase (PDK), an enzyme which represses mitochondrial respiration, forcing the cell to rely heavily on glycolysis. These alterations lead to increased glucose uptake and glycolytic flux, a phenomenon reminiscent of the Warburg effect [305–307]. This mechanism would allow maintaining the supply of lactate, which has a key role in the astrocyte–neuron interface and maintenance of neuronal function and long-term memory, as well as regulation of neuroinflammation [308]. Moreover, the cells can use the Warburg effect to support the metabolic demand and depend less on mitochondrial metabolism, so it would also have an anti-apoptotic effect [306,307] and offer a probable mechanism of cellular immortality. However, the loss of the adaptive advantage provided by aerobic glycolysis could exacerbate the pathophysiological processes associated with AD, making the brain more susceptible to Aβ-induced neurotoxicity and leading to cell death and dementia [305]. These observations may be related to what was observed in normal individuals with elevated aerobic glycolysis and high levels of Aβ deposition but without clinical manifestation of the disease [262]. Also, recent studies have revealed that the overexpression of LDH has neuroprotective effects against Aβ toxicity [309].

##### 4.1.1.3. Gliosis promotion and stable amyloid beta complexes

Increased activity of some enzymes has been documented in AD patient brain tissues [310]. Some studies associated increased HK, PFK and LDH activities with the severity and extent of neurodegeneration in AD. Although it is not clear, it is supposed that these could influence the expression of the glial fibrillary acidic protein (GFAP) and reactivation of astrocytes, functioning as a compensatory mechanism during which it would facilitate metabolic collaboration between glial cells and neurons [310]. However, gliosis induced by these glycolytic enzymes may play an important role in the pathogenesis of AD. Astrocyte reactivation could lead to proliferation of these cells and the generation of scarring in brain tissue that could affect its function [311]. Alternatively, GFAP upregulation would induce in astrocytes the release of proinflammatory markers, including nitric oxide (NO), ROS, TNF-α and IL-6, promoting a neuroinflammatory process [312]. The activation of some glycolytic enzymes could likely promote reactive gliosis and proinflammatory response in AD, both processes associated with cognitive impairment and memory loss. On the other hand, the overexpression of GAPDH and its interaction with proteins associated with AD have been related to the severity and progression of the disease [313,314]. In cerebrospinal fluid (CSF) from AD patients, high concentrations of stable GAPDH/Aβ aggregates were detected, which were directly proportional to the severity of the disease [314]. This study found that GAPDH is released into the extracellular medium by dying neurons and forms soluble covalent complexes with Aβ42, facilitating its aggregation, cytotoxicity and subsequent neuronal cell death [314] (figure 2). This suggests that detecting glycolytic enzymes, such GAPDH, in biological fluids could be a valuable tool for diagnosing AD and as well for the evaluation of its severity [315]. Alternatively, inside the cell, GAPDH in the form of aggregates can promote Aβ40 amyloidogenesis and form stable complexes that interact with the mitochondria causing disruption of the mitochondrial membrane potential and release of cytochrome c into the cytosol, leading ultimately to cell death [313] (figure 2).

##### 4.1.1.4. Oxygen transport

During AD, other peripheral cells, such as red blood cells (RBCs), are defective in glycolysis and antioxidant pathways [316,317]. Due to the absence of nuclei and mitochondria, RBCs are energetically dependent on the anaerobic catabolism of glucose via the Embden–Meyerhof pathway for the generation and storage of energy in phosphate bonds. Additionally, these cells possess a bypass in their glycolytic pathway known as the Rapoport–Luebering (RP–L) shunt, involving the enzymes bisphosphoglycerate mutase (BPGM) and bisphosphoglycerate phosphatase (BPGP). This RP–L shunt produces 2,3-bisphosphoglycerate (2,3-BPG), an allosteric modulator of Hb in RBCs. Under physiological conditions, 2,3-BPG regulates oxygen release from Hb and its delivery to tissues. The binding of 2,3-BPG decreases Hb’s affinity for oxygen [316–318]. However, alteration of activity of enzymes of glycolysis and the RP–L shunt has been reported for RBCs of AD patients [316,318]. Increases in the activities of HK, PFK, PGI, BPGM and BPGP induce a disturbance of 2,3-BPG metabolism, which leads to a significant decrease in the levels of this metabolite and consequently loss of Hb’s ability to release oxygen to tissues [316,318]. This could be related to the reduced oxygen consumption in the brains of patients with AD and neuronal dysfunction associated with hypoxia [319,320]. Also, it has been suggested that the increased activity of these glycolytic enzymes is a result of a greater demand for ATP generated through glycolysis to maintain the Na/K-ATPase pump, of which also an increased activity is found in RBCs of AD patients [317,318]. The above could support the idea that alterations in glycolysis of RBCs could be used as potential tools in the detection of AD.

##### 4.1.1.5. Chronic neuroinflammation

Another hallmark of AD is chronic neuroinflammation, and it is considered that dysfunctional glucose metabolism may contribute to this process [321,322]. Microglia, the CNS cells that function as elements of the immune system, can use both glycolysis and OXPHOS for energy metabolism. Under inactive conditions or anti-inflammatory states, the microglia depend mainly on fatty-acid oxidation and OXPHOS to support the energy demand; however, when they are stimulated and consequently activated or acquire pro-inflammatory states, a metabolic switch occurs that leads to the use of glycolysis as energy source [323,324]. This metabolic reprogramming in the microglia is associated with their function of releasing cytokines, reduction of their ability of phagocytosis and chemotaxis and changes in their morphology [323].

Different studies have shown that certain stimuli, such as the deposition and increased expression of Aβ, induce microglia to acquire a glycolytic phenotype [325,326]. Under these conditions, the preferential use of glycolysis leads to an increase in lactate levels and lactate-dependent histone modification (‘lactylation’) such as on lysine 12 of histone 4 (H4K12l), which induces transcriptional activation of some glycolytic genes (e.g. *Pkm* and *Ldha*). This creates a ‘glycolysis/H4K12la/PKM2-LDHA’ positive feedback loop and increases glycolytic activity, exacerbates microglial dysfunction and contributes to neuroinflammation in AD. Additionally, the cells with an acquired glycolytic phenotype display a decreased ability for chemotaxis and phagocytosis [326].

Paradoxically, the downregulation of glycolysis could also be associated with the neuroinflammation observed in AD [321]. In response to neuroinflammatory stimuli, the upregulation of microRNA-146a (miR-146a) expression, a small non-coding RNA that regulates immune activation and expression of proteins involved in energy metabolism, induces a reduction in glycolysis and OXPHOS in different types of CNS cells (neurons, mixed glial cells and brain endothelial cells) and promotes neuroinflammation. miR-146a can alter the expression of glycolytic enzymes within the same cell and also in neighbouring recipient cells when released in extracellular vesicles (EVs) [321]. Although it is unclear how this alteration contributes to neuroinflammation, miR-146a may serve as a link in neuroinflammation and hypometabolism. Recent studies have demonstrated the combined effects of microglia activation and hypometabolism, where both processes lead to a disruption of frontal connectivity, resulting in a positive feedback loop that leads to faster clinical deterioration [327]. Also, the release of miR-146a-EVs by microglia has been attributed to critical roles in the inhibition of neurodegeneration during depression-related pathological processes. In this sense, the miR-146a when released in EVs could function as a means of communication between glial cells and neurons [328]. All this could lead to suggest that any imbalance in glucose metabolism in microglia could have an influence on neurodegeneration during AD and that the role of this metabolic pathway may be more relevant than has been estimated in the development of this disease.

### 4.1.2. Parkinson’s disease

Parkinson’s disease (PD) is the most common form of parkinsonism (a group of neurological disorders that involves movement disturbances such as rigidity (stiffness), slowness and tremor) and it is the second most common neurodegenerative disorder, second only to AD. Currently, this disease affects more than 6 million people around the world [329]. However, its prevalence is expected to increase to between 12 and 17 million by 2040 [330]. It is estimated that around 2% of the population over 60 years of age is affected by this devastating disease [331].

In addition to the motor symptoms associated with movement (bradykinesia, tremor (usually unilateral), rigidity, postural instability), other non-motor symptoms (hyposmia, dysautonomia sleep dysfunction, psychiatric disturbances, and cognitive impairment) are observed in this disease [332,333]. Although PD is considered an idiopathic disorder, the development of this disease is mainly associated with factors such as gender (male), advanced age, family history (about 5–10% have Mendelian inheritance) and rural lifestyle (which facilitates exposure to some pesticides such as paraquat) [334–336].

The pathological hallmark of PD is depigmentation of the substantia nigra and locus coeruleus, accompanied by dopaminergic neuron loss in the pars compacta of the substantia nigra [337], in the basal nucleus of Meynert and the dorsal motor nucleus of the vagus nerve [335]. These affected areas are characterized by the presence of Lewy bodies and Lewy neurites, due to the intracellular formation of misfolded and accumulation of abnormal deposits of a protein called alpha-synuclein (α-syn) [335,338]. Although the main cause of PD is unknown, neuronal loss is attributed to inflammation, oxidative stress, protein handling abnormalities and energy metabolism alterations (reduced glycolysis, mitochondrial dysfunction and lower ATP levels are common features of PD and contribute to the pathogenesis of this disease) [335,339–341]. Currently, there are drugs that partially or transiently attenuate the symptoms of the disease; however, they do not stop or prevent neurodegeneration [342,343].

Like for AD, alterations in the glycolytic pathway have been associated with PD. Some studies have shown that impaired glucose uptake and the function and/or expression of some glycolytic enzymes are involved in the development of the disease [344–346]. In patients with Parkinson’s, hypometabolism can be observed in specific areas of the brain grey matter. Depending on the affected areas, variations have been noted in the manifestation of some symptoms such as dementia, mild cognitive impairment, or both. Additionally, it has been suggested that this hypometabolism and atrophy could be consecutive stages during the neurodegenerative process observed in PD. Hypometabolism would represent a cellular dysfunction that, when maintained, could evolve towards histological changes and neuronal death, which would finally manifest as a reduction in the volume of grey matter [347].

Therefore, poor glucose metabolism could probably be considered as a relevant initial factor of PD. This metabolic dysfunction could induce subsequent neuronal deterioration, exacerbating the metabolic imbalance and thus generating a positive feedback loop that would contribute to disease progression [348]. There have even been reports suggesting that both increased and decreased glycolysis may contribute to the pathogenesis of PD [346,349–354], which led to PD patients being classified into various subgroups based on the pattern of metabolism [355–359]. Apoptosis of dopaminergic neurons, low ATP levels and the possible development of other metabolic diseases are some of the factors that have been associated with the deregulation of the glycolytic pathway [345,346,353].

Importantly, some studies have suggested that enhancing glycolysis could be a neuroprotective mechanism in PD. This may be achieved using glycolysis-enhancing drugs, which modulate or enhance the activity of some enzymes related to the generation of ATP, such as PGK-1, thereby stimulating glycolysis, increasing cellular ATP levels and then slowing or preventing neuron loss [360–363]. On the other hand, studies have reported an age-dependent significant increase in PGK activity in red blood cells from patients with PD. This alteration of PGK activity of PD patients was significantly greater than that of the control group in participants aged 65 years or younger and was negatively associated with striatonigral degeneration. This has led to PGK being considered as a diagnostic biomarker for PD and a tool to therapeutically monitor treatments that modulate the activity of this PGK enzyme [364]. However, more in-depth studies have been suggested on these findings [362,365].

#### 4.1.2.1. Apoptosis of dopaminergic neurons

Dysregulation of some glycolytic enzymes has been linked to PD. Increased HK-2 and LDH-A expression induces apoptosis of dopaminergic neurons (DpNs) by promoting excessive lactate production [345] (figure 2). Although lactate can be used by DpNs as a bioenergetic metabolite, its accumulation induces activation of the AMPK/AKT/mTOR pathway and subsequent apoptosis of DpNs [345]. Apparently, lactate can activate the phosphorylated form of the AMP-activated kinase (AMPK). Once AMPK is activated, it could negatively regulate the TOR pathway, subsequently activating the AMPK/AKT/mTOR pathway [345], which has been related to the apoptosis of dopaminergic neurons [366] (figure 2). Notably, lactate-triggered AMPK activation may influence the accumulation of α-syn and decreased neurite outgrowth in dopaminergic neuroblastoma cells [367]. On the other hand, the accumulation of lactate can lead to intracellular acidification, favouring the aggregation of α-syn and subsequent interactions with mitochondria, via cardiolipin, which possibly leads to alterations in the physiology and turnover of these cellular structures [368,369] which ultimately leads to mitochondrial dysfunction and a drop in ATP levels.

#### 4.1.2.2. Alteration of protein homeostasis (proteostasis)

Some studies have provided support for the notion of glycolysis acting as a conserved functional node at the intersection of proteostasis and neurodegeneration [370]. In *C. elegans* studied as a model, it was found that the enzyme PGI can act as a potent modifier of α-syn misfolding and neurodegeneration of DpNs (figure 2), while in *D. melanogaster* the mutation of the gene induced an increase in neuroinflammation, alteration of homeostasis and induction of degeneration of these DpNs. Likewise, in mice, the loss of this functional enzyme resulted in α-syn accumulation and neurotoxicity in primary cortical neurons. Apparently, PGI regulates the folding of endogenous metastable proteins via glycolysis [370]. Although the mechanism of neuroprotection is unclear, upregulated PGI in tumour cells can act as a ligand of the E3 ubiquitin-protein ligase hrd-like protein 1 (HRDL-1)/autocrine motility factor receptor (AMFR) in the AMF pathway, mitigating endoplasmic reticulum (ER) stress and cellular apoptosis [371]. Alternatively, HRD1, functioning as a homologue of the HRD family of E3 ligases, regulated 3-hydroxy-3-methylglutaryl coenzyme A (HMG-CoA) reductase by modifying it through ubiquitination, directing it for degradation by proteasomes [372,373], which has led to the suggestion of a potential relationship between lipid biosynthesis and α-syn toxicity in PD [370]. Lipid binding is known to modulate the initial aggregation of α-syn and neurotoxicity [374,375]. It is thus possible that alteration of glucose metabolism may modulate α-syn toxicity through a mechanism involving the elevation of levels of certain lipids that bind to α-syn and facilitate the toxic oligomerization process [370]. Also, alterations in the glycolytic pathway could impact post-translational modifications (PTMs) of α-syn. Some PTMs, such as glycation and glycosylation, influence the ability of α-syn to bind to lipids, aggregation and toxicity of α-syn [375]. Nine residues of α-syn have been documented as potential sites of glycosylation, and this modification reduces aggregation and toxicity of α-syn [376]. The neuroprotective function associated with PGI-1 has also been documented in an animal model of ataxia, modulating the neuroprotective properties of oxidation resistance protein 1 (Oxr1), a key player in the regulation of oxidative stress and glucose metabolism in the brain [377].

#### 4.1.2.3. Positive feedback with other pathologies

It has been documented that glycolysis is directly regulated by genes associated with PD [378]. Overexpression of α-syn not only directly inhibits the activity of some glycolytic enzymes, such as GAPDH, and consequently affects glucose metabolism, but also has a regulatory effect on these enzymes through modifications of their expression [379]. Other proteins such as PARKIN modulate the glucose metabolism through the ubiquitination of PKM1 and PKM2, resulting in decreased enzymatic activity [380]. Notably, several studies have demonstrated the direct interaction of other glycolytic enzymes with α-syn. Some enzymes such as ALD-A, PGK-1, PGM-1 and ENO-2 can be sequestered by amyloid-like structures such as a-syn fibrils [381,382]. The interaction of PKM2 with other PD biomarker proteins, such as the deglycase protein (DJ-1, also known as protein 7), has also been observed [383]. It is noteworthy that around 144 proteins could possibly form aggregates with α-syn and DJ-1, of which 6% are related to metabolism [383]. All of this could suggest that the mechanism by which a-syn and other PD-associated proteins promote dysfunction could be directly related to the inhibition of glycolysis and development of diseases associated with glucose metabolism, taking into account how glycolysis and insulin secretion are coupled to the energy state (ATP/ADP ratio) in β-cells, where ATP levels play a relevant role in activating a cascade of events that ultimately lead to the cellular release of insulin [384].

Previously, a positive association has been observed between type 2 diabetes mellitus (T2DM) and PD, where T2DM was reported as a risk factor for PD and playing a role in its progression [385]. Similarly, in murine models, long-term hyperglycaemic conditions, T1/2 DM, have been shown as a potential risk factor in PD pathogenesis [386]. Neuroinflammation is considered one of the main processes linking hyperglycaemia and PD. High blood glucose concentrations can saturate mitochondrial respiration in endothelial cells, astrocytes and pericytes, leading to ROS production and consequently oxidative stress (figure 2). During oxidative stress-induced mitochondrial dysfunction, an increase in the expression of heat shock protein 60 (HSP60) occurs. Excess HSP60 translocates to the cell membrane where it binds to pattern recognition receptors (PRRs), triggering inflammatory signalling cascades that stimulate the release of pro-inflammatory mediators, enhancing inflammation in neuronal cells. In parallel, HSP60 can be released by EVs and travel to neighbouring astrocytes, binding to their cell membrane PRRs, amplifying the inflammatory response [387]. Alternatively, ROS and pro-inflammatory cytokines can disrupt the integrity of the BBB and alter the homeostasis of the brain environment, leading to microglial activation and neuroinflammation [386]. Hyperglycaemia could also induce morphological alteration of astrocytes, caused mainly by changes in the expression of proteins of the cytoskeleton, such as GFAP and vimentin [388].

It has also been documented that in patients with DM2 there is an increased accumulation of phosphorylated α-syn in pancreatic β cells and the brain [389,390] (figure 2). This could be related to the observation in other studies that α-syn may have a direct or indirect regulatory role in glucose metabolism in the brain and peripheral tissues. The α-syn in blood plasma regulates glucose uptake in peripheral tissues (such as adipose tissue, pancreatic islets and skeletal muscle) through the LPAR2/Gab1/PI3K/AKT transduction pathway, playing a relevant role in the maintenance of normal glucose homeostasis [391,392]. Like what occurs in neurons, where α-syn is associated with exocytic neurotransmitter release, in pancreatic islets α-syn interacts with ATP-sensitive potassium (K_ATP_) channels at insulin-secretory granules, acting as an inhibitor of the secretion of this hormone, but that glucose stimulation of insulin secretion can override [393]. In this context, α-syn interacts with secretory vesicles to slow their progression or prepare them through the secretory pathway to exocytic sites for release [393]. It is important to highlight that GAPDH is also part of the proteome of insulin granules, and is one of the proteins that interacts with their K_ATP_ channels [394]. All of this could support the idea that the expression of mutated forms of a-syn could have various effects on insulin secretion or glucose uptake in peripheral tissues. For example, an excessive blockade, which could lead to conditions that lead to cellular stress, a common event of some forms of diabetes or PD [393].

In addition, α-syn interactions with insulin-secretory granule components could involve the formation of Lewy body-like pancreatic inclusions, promote fibril formation of the complex α-syn/IAPP (IAPP, islet amyloid polypeptide, also known as amylin, involved in the regulation glucose homeostasis) which would contribute to β-cell apoptosis development of diabetes [390,395]. Also, the accumulation of a-syn and IAPP causes complement system activation, as measured by deposition of the C5b-9 complex, and subsequent stimulation of the innate immune response and chronic inflammation [396]. This could be related not only to the observed association between T2DM and some NNDs, such as PD [397,398], but as well to the linkage between the activation of the innate immunity system, chronic inflammation and T2DM [399,400]. Also, all this could suggest a positive feedback loop between long-term hyperglycaemia and the activation of associated pathogenic mechanisms.

### 4.2. Cancer

Reprogramming of cell metabolism is one of the hallmarks of tumour cells [401,402]. During the cell transformation process occurs a switch from the use of OXPHOS to that of aerobic glycolysis as the main process for energy generation. Although the mechanisms involved in the promotion of this dependence on high glycolytic rates in tumour cells, even under optimal oxygen conditions, are not known, overexpression of enzymes, transcription factors and some signalling molecules, including oncogenes and tumour suppressors, could be involved in the attenuation of mitochondrial function and triggering of the Warburg effect, a key factor for tumorigenesis and malignancy progression [402,403].

#### 4.2.1. Cell proliferation

**Figure 3.**
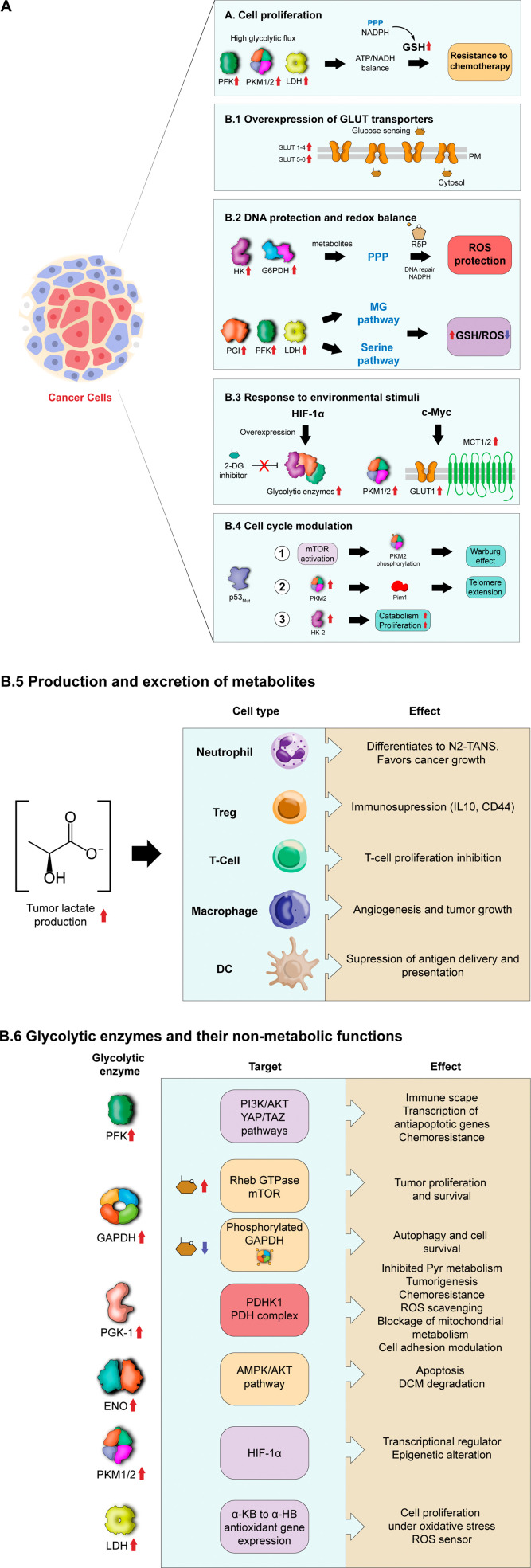
Dysregulation and effects of glycolytic enzymes in cancer. (*a*) Cell proliferation. The upregulated PFK, PKM1/2 and LDH sustain a high glycolytic flux. Consequently, the GSH concentration increases to promote resistance to chemotherapy. (*B.1*) Overexpression of GLUT transporters. This overexpression promotes glucose sensing and the migratory property of cancer cells induced by glucose. (*B.2*) DNA protection and redox balance. The upregulation of glycolytic enzymes affects other metabolic pathways. Increased expression of HK and G6PDH feeds the PPP with G-6-P to produce key metabolites that protect against ROS. The upregulation of enzymes such as PGI, PFK and LDH contributes to the redox balance (GSH/ROS) that eventually protects the cancer cells. (*B.3*) Response to environmental stimuli. In cancer cells, transcription factors HIF-1α and c-Myc modulate the expression of glycolytic genes under hypoxic and normoxic conditions, respectively. Resistance to 2-DG is promoted by the increased expression of glycolytic enzymes induced by HIF-1α, while c-Myc induces the expression of GLUT and MCT1 and 2 transporters to regulate lactate concentrations in tumour cells. (*B.4*) Cell cycle modulation. In cancer cells, mutant p53 favours the translocation of GLUT1 to the membrane. The activation of the mTOR pathway induces the phosphorylation of PKM2 which leads to induction of the Warburg effect in cancer cells. (*B.5*) Production and excretion of metabolites. The high glycolytic flux of cancer cells leads to increased lactate production and excretion. This lactate modulates the response of several immune cells involving the induction or depletion of metabolites or signalling molecules. In this way, lactate affects the migration and differentiation of neutrophils, becoming cells with reduced phagocytic activity, diminished production of ROS and changes in adhesion molecules that provide a better environment for cancer metastasis. In macrophages, the epigenetic changes caused by lactate lead to cell polarization (M2 macrophages) that promotes angiogenesis and tumour growth. On the other hand, in T-cells, lactate induces the release of immunosuppressive cytokines (e.g. IL-10) in the case of Tregs or even promotes the inhibition of proliferation as occurs with other T-cells, while in dendritic cells (DC), lactate leads to alteration of the metabolic state, migration, differentiation and to the ability to process and present antigens to other immune cells. (*B.6*) Non-metabolic functions of glycolytic enzymes in cancer cells. The chart summarizes the most important targets and effects of the overexpression of glycolytic enzymes. The moonlighting functions of glycolytic enzymes are focused on the regulation of gene expression as a response to environmental stimuli and the activation and modulation of specific signalling pathways that favour tumorigenesis. Phosphofructokinase (PFK), glyceraldehyde-3-phosphate dehydrogenase (GAPDH), phosphoglycerate kinase (PGK), enolase (ENO), pyruvate kinase M isoforms (PKM), lactate dehydrogenase (LDH) and triose-phosphate isomerase (TPI).

The main mechanism that allows cancer cells to maintain a high glycolytic flux is the overexpression of some glycolytic enzymes such as PFK, ENO-3, PKM2 and LDH, and one of the isoforms of the bifunctional enzyme 6-phosphofructo-2-kinase/fructose-2,6-bisphosphatase (also known as PFK-2) that produces F-2,6-BP, the potent activator of the glycolytic PFK [404] (figure 3*a*). These enzymes are critical in regulating and enhancing cell glycolysis [34,405,406], and regulating the balance between glycolytic ATP generation and biosynthetic needs for proliferating cells [407]. The use of the glycolytic pathway by tumour cells could be related to the increased rates of ATP production through this pathway and that of the generation of glycolytic intermediates to meet the biosynthetic needs of rapidly proliferating cells. NADPH, also required for the increased need of biosynthesis, is produced in different metabolic routes, most prominently the PPP, linked to glycolysis through its intermediate G-6-P. In addition, when tumours are subjected to chemotherapeutic agents, their cells may require an increased production of reduced glutathione (GSH) to deal with the stress. This will in turn require an additional production of NADPH, thus necessitating a further increase in glucose consumption (figure 3*a*).

#### 4.2.2. Resistance to radiation and chemotherapy

In addition to supporting the metabolic demands of malignancy, a high glycolytic flux in tumour cells has also been reported in relation to chemo- or radio-resistance [408–410]. An increase in the expression of the glycolytic pathway (transporters and enzymes), leading to a higher glycolytic flux can contribute to development of resistance against the treatment through an increase in DNA repair, immunosuppression in the tumour microenvironment and apoptosis resistance by enhanced autophagy [409] (figure 3*B.1–B.3*).

##### 4.2.2.1. Overexpression of GLUT transporters

Transporters involved in the uptake of glucose into cells, class I (GLUT1-4) and class II (GLUT5-6) transporters, have been associated with the process of carcinogenesis, the increasing aggressiveness and invasiveness of tumours and the induction of resistance against antitumour therapies [411,412]. Similarly, class III glucose transporters such as GLUT12, an insulin‐responsive transporter, are also overexpressed in cancer cells [413]. Although the mechanisms involved in the resistance induced by GLUT transporters are poorly understood, the expression level of several of these transporters has been correlated with glucose sensing and acquisition of glucose-induced migratory properties of tumour cells (figure 3*B.1*).

##### 4.2.2.2. DNA protection and redox balance

It has been observed that cancer cells, by substantially enhancing the rate of glucose influx via activating enzymes involved in the reactions converting glucose to PEP (from HK to ENO), and the attenuation of the last step, the conversion of PEP to pyruvate, by expression of the PKM2 isoform of which the velocity can be allosterically regulated, allow a greater flow of glycolytic intermediates to many biosynthetic routes. This would provide not only molecules necessary for the proliferation of tumour cells but also for their survival through DNA repair, inhibition of DNA damage (caused by ROS) and decreased chromatin remodelling [118,409,414].

The overexpression of HK and G6PDH would provide metabolites to feed the PPP, which generates ribose-5-phosphate, a precursor in nucleotide synthesis and DNA repair, and NADPH, which plays a key role in biosynthetic processes and the protection against ROS [409]. Increased expression of PGI would promote the activation of the methylglyoxal (MG) pathway, because when PGI’s product, F-6-P, accumulates, it could be converted into DHAP that feeds the MG pathway [409,415]. This pathway involves a series of steps that regulate the intracellular NADPH content, a key factor that manipulates ROS hormesis [416]. Facilitation of tumour growth and metastasis is also attributed to this pathway [415]. Besides, the MG interaction with amino groups of proteins leads to the formation of advanced glycation end products (AGEs). The accumulation of AGEs has been documented in some tumours [417,418]. MG-derived advanced glycation of specific proteins, such as heat-shock protein 27 (HSP27), reduces DNA damage and is considered a possible mechanism by which apoptosis is evaded in some types of cancer [415].

The enhancement of glycolytic enzymes such as the ALD-A isoform and PGK, leading to intermediate 3-phosphoglycerate (3-PGA) accumulation and subsequent increase of serine synthesis [409,419], is also involved in maintaining a balance of the redox state of the cell [409]. The conversion of 3-PGA into serine occurs through three sequential enzymatic reactions (catalysed by phosphoglycerate dehydrogenase (PHGDH), phosphoserine aminotransferase (PSAT1) and phosphoserine phosphatase (PSP)). Subsequently, serine is converted to glycine by enzyme serine hydroxymethyltransferase 1 (SHMT1). Notably, the conversion of serine to glycine generates 5,10-methylenetetrahydrofolate (CH2-THF), which is then used in folate (FC) synthesis and the methionine cycle (MetC), important for DNA/histone methylation and producing reducing power against ROS. Moreover, FC is an additional source to generate NADPH, necessary for several metabolic processes [414,420,421]. Another relevant aspect of de novo serine synthesis is that this amino acid can be converted into glycine, an important source for glutathione (GSH) biosynthesis, the main antioxidant factor in the defence against ROS [422]. GSH synthesis occurs through an ATP-dependent two-step enzymatic process, with the first step being catalysed by glutamate-cysteine ligase (GCL), which conjugates cysteine with glutamate, generating γ-glutamylcysteine. The second step is catalysed by GSH synthase, which adds glycine to γ-glutamylcysteine to form γ-glutamylcysteinylglycine or GSH [423]. In addition to the above, it has also been suggested that the de novo synthesis of serine in tumour cells could serve as a link between mTOR signalling and DNA methylation, favouring tumour growth [424].

In tumour cells, the serine synthesis pathway thus maintains the cellular redox balance by supporting glutathione synthesis, decreased ROS accumulation and proliferation, while the MG pathway helps maintaining the redox state and promotion of tumour growth and metastasis. This could lead to the suggestion that tumour cells adopt aerobic glycolysis to avoid the metabolic generation of ROS [425,426] (figure 3*B.2*).

##### 4.2.2.3. Response to environmental stimuli

Chemo- and radio-resistance in tumour cells associated with glycolysis is related to responses to either environmental stimuli, derived from the microenvironment (e.g. hypoxia or pseudohypoxia), or stimuli that are intrinsic to the cell (e.g. oncoproteins) [410,427]. Some transcription factors (such as hypoxia-inducible factor-1α (HIF-1α) and v-Myc avian myelocytomatosis viral oncogene homologue (c-Myc)) and oncoproteins have been described as master inducers of glycolysis in cancer [36]. These transcription factors promote the expression of glucose transporters and glycolytic enzymes, through their binding to a consensus sequence located within the promoter region of the genes encoding these proteins [36,428] (figure 3*B.3*).

The upregulation of HIF-1α plays an essential role in the regulation of glycolysis under hypoxia [429] and adaptation to the hypoxic microenvironment [430]. HIF-1α promotes the expression of glucose transporters (GLUT1 and GLUT3) and at least eight of the ten glycolytic enzymes (HK, PFK, ALD-A, GAPDH, PGK, ENO, PKM and LDH-A) [431–433] (figure 3*B.3*), allowing an increased flux through the pathway and maintenance of cellular ATP production under hypoxic conditions and promoting in turn an increase in cell proliferation and angiogenesis [433]. Notably, it has been shown that HIF-1 confers resistance to the glycolysis inhibitor 2-deoxy-d-glucose (2-DG) and other glycolysis inhibitors. The overexpression of the glycolytic enzymes, promoted by HIF-1α, results in resistance to 2-DG as antitumour drug, since higher concentrations of 2-DG are required to inhibit glycolysis [434].

For its part, c-Myc directly activates the transcription of almost all glycolytic genes at normal oxygen levels [435,436], and unlike HIF-1α was also shown to promote the preferential production of PKM2 over PKM1 by changing exon splicing of the PKM transcript and subsequent promotion of aerobic glycolysis [437]. Additionally, it can also up-modulate the expression of GLUT1 [438], as well as that of the monocarboxylate transporters, MCT1 and MCT2, through transcriptional repression of microRNAs (miR-29a and miR-29c), to control toxic levels of lactate within tumour cells [439] (figure 3*B.3*). Alterations in other proto-oncogenes such as H-RAS, v-SRC and AKT induce increased rates of aerobic glycolysis indirectly through increased expression of HIF-1α and thus its targets [440–442], as well as some angiogenic factors (such as vascular endothelial growth factor (VEGF) and erythropoietin (EPO) [443]), required for survival and tumour growth.

Generally, increased expression of glycolytic enzymes is associated with chemoresistance. However, downregulation of glycolytic enzyme expression has also been linked to chemoresistance and poor prognosis in some cancer types [444–448]. Various studies found that mRNA and protein expression levels of PGAM-1 are downregulated in methotrexate-resistant human breast cancer cells. The authors suggested that an aberrant glucose metabolism could play a relevant role in multidrug resistance (MDR) in breast cancer, although the mechanism is unknown [444]. Other enzymes, such as PGI, PGK-1 and ENO-2, were found to be downregulated in an epidermal growth factor receptor (EGFR)-mutant non-small-cell lung cancer (NSCLC) with advanced resistance to EGFR tyrosine kinase inhibitors (TKIs), such as erlotinib. The mechanism of resistance involves metabolic reprogramming, characterized by global bioenergetics suppression including a reverse Warburg effect, directed by TGFβ2 signalling. This reprogramming leads the cells to a proliferative-metabolic quiescent state [446]. It should be noted that, over time, resistant quiescent tumour cells could become the origin of a cancer resistant to chemotherapy. Furthermore, such cells could go undetected by clinical ^18^F-fluoro-2-DG-PET scanning due to their suppressed glucose metabolism [446]. Likewise, downregulation of glycolysis has been linked to resistance to cisplatin in ovarian cancer cell lines. The expression of enzymes such as PGI, ALD, PGK and PKM2 was downregulated in cisplatin-resistant cell lines, indicating that drug resistance in ovarian cancer could be associated with a decrease in glycolysis. However, the significance of this downregulation is still poorly understood [445]. All of this could support the idea that the up- or downregulation of glycolysis has a relevant role in the development of cancer and that glycolytic enzymes could fulfil different cellular functions, depending on the context or type of cancer.

### 4.2.3. Cell cycle modulation

Oncoproteins such as p53 play roles in tumour suppression, regulating the expression of genes involved in cellular processes such as cell cycle arrest and apoptosis [449]. Regulation of various energy-metabolic pathways is one of the important functions of p53 [450]. In healthy conditions, it represses glycolysis and the Warburg effect, through the regulation of genes involved in this metabolism [450,451]. However, mutations in gene *p53* are very common in various types of cancer and are associated with diverse prognostic values [452]. Many of them are nonsense mutations, leading to the production of a complete protein with a single substitution and gain-of-function (GOF) [453]. Some studies have revealed that GOF-mutant p53 proteins (mutp53) drive glucose uptake and the Warburg effect in tumour cells, by promoting GLUT1 insertion in the plasma membrane, via activation of the RhoA/ROCK signalling, so contributing to cancer progression [451] (figure 3*B.4*). Mutants such as mutp53(R175H and R273H) can also promote glycolysis by PKM2 phosphorylation through the involvement of mTOR signalling. In tumour cells, overexpression of death-associated protein kinase 1 (DAPK1) mediated the disruption of the interaction between tuberous sclerosis complex proteins 1 and 2 (TSC-1/TSC-2), resulting in the activation of the mTOR pathway. Subsequently, mTOR phosphorylates PKM2, promoting its stabilization in its homodimeric form, and favouring the Warburg effect [454] (figure 3*B.4*). Mutp53(N340Q/L344R) can also promote hepatocarcinogenesis through the upregulation of PKM2 by forming a complex with the long non-coding cancer-upregulated drug-resistant (CUDR) RNA, which binds to the promoter region of the *pkm* gene and stimulates the expression, phosphorylation and formation of PKM2 tetramer [455]. PKM2 binds to histone H3, favouring the phosphorylation of its threonine at position 11 of (pH3T11). This phosphorylation of H3T11 blocks the binding of histone deacetylase 3 (HDAC3) to the acetylated H3K9 (H3K9Ac), preventing its deacetylation and stabilizing this modification. Simultaneously, there is a reduction in the trimethylation of lysine 9 on H3 (H3K9me3) and an increase in its monomethylated form (H3K9me1). The combination of H3K9me1 and heterochromatin protein 1α (HP1α) promotes the formation of a complex that binds to the promoter region of proto-oncogene *pim1*, enhancing its expression [455]. Pim1 is a serine/threonine-protein kinase involved in the maintenance of telomere length by regulating the expression of genes such as TERT (telomerase) and HOX antisense intergenic RNA (HOTAIR) lncRNA, associated with telomere lengthening in cancer cells [455] (figure 3*B.4*).

Mutp53 can also promote the upregulation of other glycolytic enzymes such as HK-2. In AS-30D hepatoma, mutations peripheral to the DNA-binding domain in mutp53(Gly105/Glu256) induce binding of novel *cis*-elements within the HK2 promoter and promote increased expression of the enzyme, facilitating high rates of glucose catabolism, and subsequently a rapid cell proliferation [456]. In this sense, glycolytic enzymes could be a link between the loss of cell cycle control and a high glycolytic phenotype in tumour cells.

### 4.2.4. The production and excretion of metabolites

The expression and activation of some glycolytic enzymes are also key to the formation of an immunoprotective niche, thus promoting the proliferation, maintenance and progression of malignant cells, through either the production of inhibitory metabolites and/or depletion of essential metabolites in the microenvironment [457]. The overproduction of some metabolites in this pathway, such as lactate, function as signalling molecules that modulate immune cell functions in the tumour microenvironment (TME) (figure 3*B.5*). Lactate is known to cause vascular permeability, and its production by tumour cells also causes mobilization of tumour-associated neutrophils (TANs) [458], which may have a dual role in tumour growth depending on their polarization states [459,460]. Additionally, lactate also influences the differentiation of neutrophils into their N2 phenotype (N2-TANs) or pro-tumorigenic neutrophils [458,461]. N2-TANs are characterized by a decreased neutrophil activity and cytotoxicity. These N2-TANs have low phagocytic activity, an impairment in ROS production and expression of high levels of integrin β2 and arginase 1 (Arg1). These cells promote tumoural growth and metastasis through multiple mechanisms including T-cell suppression and production of immunosuppressive cytokines, angiogenic factors (such as vascular epithelial growth factors (VEGF)) and matrix-degrading proteases (matrix metallopeptidase-9 (MMP-9) and neutrophil elastase (NE)) [461,462] (figure 3*B.5*). The presence of N2-TANs in TME is associated with poor prognosis in various types of cancer [461]. In addition, TANs are glycolytic cells [463], so it is very likely that lactate has an impact on the metabolic profile which subsequently leads to an alteration of the apoptosis and effector activities (e.g. phagocytosis and induction of neutrophil extracellular traps (NETs)) of these cells [464,465], which play an important role in tumour invasion and migration [465].

The production and excretion of high concentrations of lactate are also associated with the recruitment of immune cells and promotion of differentiation of immunosuppressive cell types, such as regulatory T cells (Treg), alternatively activated (M2) macrophages and myeloid-derived suppressor cells (MSDC) which further suppress antitumour immune responses [464,466]. Studies of melanoma have shown that tumours avoid destruction through the metabolic support of Treg populations. Lactate can stimulate Treg cells, via G-protein-coupled receptor GPR81, enter as a metabolite via monocarboxylate transporter 1 (MCT1) and be consumed by them, and serve as substrate for the synthesis of upstream glycolytic intermediates through gluconeogenic reactions [467]. However, this consumption of lactate enhances the immunosuppressive activity of these lactate-avid Treg cells, through the production of the immunosuppressive cytokine IL-10, the stem cell factor CD44 and the proangiogenic co-receptor Nrp1 [467] (figure 3*B.5*). Notably, these populations of lactate-avid Tregs are the result of a mechanism that allows these cells to be active even in times of starvation and/or nutrient deficiency, by metabolizing lactate to fuel their metabolic demand [468].

Similarly, lactate has modulatory effects on other cells of the immune system, such as macrophages, dendritic cells and natural killer cells [469]. Unlike Tregs, high extracellular concentrations of lactic acid disrupt the lactate and [H^+^] gradients between T cells and their environment, reducing MCT1-mediated export of lactic acid from the T cells and inhibiting their proliferation. In macrophages, lactate acts as an epigenetic modulator and induces the polarization of M2 macrophages through epigenetic reprogramming. It can bind directly to lysines of some histones and cause a PTM known as lysine lactylation (Kla) [470], which facilitates the transcription of genes associated with M2 polarization (such as *Arg1*) and inflammation-independent pathways involving adrenomedullin and vascular epithelial growth factors (VEGFs), as well as IL-10, programmed death ligand 1 (PD-L1) and TGFβ, which support angiogenesis and tumour growth [469,471] (figure 3*B.5*). In dendritic cells (DCs), lactate also acts as a signalling molecule through its binding to G protein-coupled receptor 81 (also known as hydroxy-carboxylic acid receptor) (GPR81/HCAR1). Activation of this receptor promotes alterations in migration, differentiation and metabolism, as well as reduction of cAMP, IL-6 and IL-12 levels, and suppression of antigen delivery and presentation (Ag) [469,472] (figure 3*B.5*). Notably, lactate can act as a source of energy by shuttling between different cell populations, and regulate multiple signalling pathways, such as the one that involves the mammalian target-of-rapamycin (mTOR) [473]. Activation of the mTOR pathway increases the expression of HIF-1α and c-Myc, which promote the expression of GLUT1 and some glycolytic enzymes, such as HK-2, PKM2 and LDHA. For its part, PKM2 can also modulate the mTORC1 pathway by inhibiting proline-rich AKT substrate 1 (PRAS40), a negative regulator of the mTORC1 loop [474,475]. All these events facilitate the production of lactate, thus providing a positive feedback.

### 4.2.5. Glycolytic enzymes and their non-metabolic functions in tumorigenesis

To meet the nutrient demands of tumour cells, many of the glycolytic enzymes also contribute to tumorigenesis through crucial non-metabolic functions (figure 3*B.6*). Some of these functions are related to the regulation of gene expression and interaction with proteins outside metabolism [410,448]. In tumour cells, some enzymes such as PFK, GAPDH, PGK and PK accumulate and translocate to the nucleus and other intracellular locations in response to stimuli, where they can regulate gene expression and interact with other proteins not involved in metabolism. Many of such moonlighting functions of these glycolytic enzymes are due to PTMs which alter their activity and subcellular localization [476–478]. Also, many of these non-metabolic functions are carried out by embryonic isoforms of these glycolytic enzymes [410].

*PFK* is considered an enzyme with a key role in the regulation of cancer development and progression, since its overexpression leads to the creation of the ‘Gordian Knot’, through the activation of the PI3K/AKT and YAP/TAZ pathways [479]. In cancer cells, F-1,6-BP promotes glycolysis and activates PI3K/AKT through several pathways, by Ras and son of sevenless homologue 1 (Sos1) induction [480] and activation of receptors associated with the EGFR oncogenic pathways [481]. PI3K/AKT, once activated, negatively regulates Hippo pathway kinases (Ste20-like serine/threonine kinases 1/2 (MST1/2) and large tumour suppressor protein serine/threonine kinases 1/2 (Lats1/2)) through phosphorylation and subsequent dissociation of the Hippo central complex and consequent inactivation of LATS. Inhibition of the Hippo pathway promotes the nuclear localization of YAP/TAZ proteins and their stable binding to TEA-domain containing transcription factors (TEAD), forming YAP/TAZ-TEAD complexes. Along with other transcription factors (including AP-1, E2F, MYC), these complexes promote the transcription of antiapoptotic genes, as well as those related to plasticity, migration and drug resistance [479] (figure 3*B.6*).

In addition to activating YAP/TAZ, PI3K/AKT can stimulate translocation of the low-active dimeric PKM2 to the nucleus, where it acts as a coactivator by binding to transcription factors such as HIF-1α and STAT3, thereby stimulating the transcription of genes of several glycolytic enzymes (PFK, PKM2, LDH-A). Also, PKM2 may promote β-catenin stabilization and subsequent transcriptional activation of YAP/TAZ, which in turn mediates the transcription of genes associated with both cell-cycle progression and glycolysis [479]. Additionally, reports indicate that the expression of nuclear PKM2 contributes to cancer stem cell (CSC)-like phenotypes by upregulating c-Myc and cyclin D1 as a co-activator [482].

It has also been reported that PI3K/AKT can promote the activation of 3-phosphoinositide-dependent protein kinase-1 (PDK1) and subsequent inhibition of pyruvate dehydrogenase (PDH) [483]. As a result, pyruvate does not feed the TCA cycle but is oriented towards lactate production by LDH-A. This lactate, secreted in the TME, inhibits the immune response and favours the establishment of an acidic pH, which negatively affects the action of many anticancer agents (including immunotherapies and mTOR inhibitors) [483–485] (figure 3*B.6*).

For its part, F-1,6-BP can bind to the epidermal growth factor receptor (EGFR) and successively induce acetylation (on K395), translocation to the plasma membrane and phosphorylation (on Y64) of the platelet isoform of PFK (PFKP). Phosphorylated PFKP binds to the N-terminal SH2 domain of the regulatory subunit of PI3K (p85α), which in turn results in PI3K/AKT activation and enhances aerobic glycolysis [479,486]. This EGFR/F-1,6-BP pathway not only may be involved in gene transcription, but also favours lactate production, which leads to inhibition of local cytotoxic T-cell activity and immune escape [481] (figure 3*B.6*). Notably, F-1,6-BP can inhibit mitochondrial OXPHOS and citrate synthesis, which prevents potential PFK inhibition. This maintains aerobic glycolysis and increases lactate production, resulting in inhibition of the immune response, as well as extracellular acidification and drug resistance [479,487].

It has also been documented that alterations in the subcellular localization of PFK could enhance tumour development. In the presence of glucose, PFK has also an ectopic nuclear expression and stimulates YAP/TAZ/ TEAD, favouring the expression of genes regulated by this complex [479,488] (figure 3*B.6*). All these observations suggest roles of PFK in cancer: activating different signalling pathways, directly and indirectly, by functioning as glycolytic enzyme and/or regulating the function of pro-tumour pathways.

Furthermore, *GAPDH* could stand for a link between energy metabolism and RNA biogenesis [489]. At low glucose concentration, GAPDH binds and sequesters the small GTPase Rheb, whereas under conditions of abundant glucose Rheb is released from GAPDH, because the binding is destabilized by glyceraldehyde-3-phosphate. Rheb then binds to mTOR and allosterically activates its kinase activity. The Rheb/mTORC1 complex stimulates the activity of transcription factors and proteins such as HIF-1α, STAT3, PPAR, SREBP and S6K1, facilitating the activation of various processes that, by different mechanisms (metabolic reprogramming, providing energy, sustaining metabolism and division) all support tumour cell proliferation and survival (figure 3*B.6*). Among these processes are the Warburg effect, anabolic pathways (PPP, fatty-acid and cholesterol synthesis), apoptotic protection, as well as autophagy, mitophagy and angiogenesis [476,490]. In response to glucose starvation, GAPDH is phosphorylated by AMPK, leading to its translocation into the nucleus where it directly triggers Sirt1 deacetylase activity, displaces Sirt1’s repressor and promotes autophagy to increase cell survival [491] (figure 3*B.6*). Additionally, GAPDH can act as a noncanonical RNA-binding protein, through recognition of the AU-rich region of its mRNA 3′-UTR, and facilitate mRNA stability and expression of genes for proteins playing important roles in cancer, such as GLUT1, colony-stimulating factor-1 (CSF-1) and CCN family member 2/connective tissue growth factor (CCN2/CTGF), involved in tumour phenotype, angiogenesis and metastasis [492,493] (figure 3*B.6*).

Like other glycolytic enzymes, *PGK-1* does not only participate in glucose metabolism but can also do so in various biological processes linked to tumorigenesis, including radioresistance [494] (figure 3*B.6*). In response to environmental conditions, PGK can act as a kinase and co-activator of transcription factors [101,495,496]. In brain cancer, hypoxia and glutamine deprivation, the acetyltransferase ARD1 interacts and acetylates PGK-1 at K388. Acetylated PGK-1 induces phosphorylation at S30 of Beclin-1 and subsequent formation and recutting of the Beclin-1–VPS34 complex, necessary for initiation of autophagy [497] (figure 3*B.6*). In endometrial carcinoma, PGK enhances the chemoresistance by triggering the HSP90/ERK pathway. Cisplatin exposure induces PGK-1 expression and subsequent stimulation of heat shock protein 90 (HSP90) activity and HSP90/ERK pathway activation, promoting expression of DNA repair-associated proteins (FOSL1, c-JUN and POLD1) and enzymes involved in DNA methylation (DNMT1, DNMT3A and DNMT3B) [498]. Alternatively, it has been found that in leukaemia PGK can protect tumour cells from 1,4-benzoquinone-induced toxicity by acting as a ROS scavenger and inhibiting ROS-induced apoptosis [499] (figure 3*B.6*). Importantly, resistance to radiation and drugs (such as 5-fluorouracil, mitomycin-C, vincristine, adriamycin, doxorubicin and mitoxantrone) is attributed to overexpression of PGK-1 and activation of some common pathways such as autophagy, activation of pathways involving HSP90 and protection against ROS [495].

It has also been found that activation of EGFR and expression of RAS GTPases (such as K-Ras and B-Raf) induce PGK-1 translocation to the mitochondria following an ERK kinase-dependent phosphorylation of PGK-1 at S203. Phosphorylated PGK-1 is imported into mitochondria through its interaction with the TOM complex and peptidyl-prolyl *cis*/*trans* isomerase (PPIase) PIN1. Within the mitochondria, PGK-1 functions as a protein kinase to phosphorylate and activate pyruvate dehydrogenase kinase 1 (PDHK-1), which further leads to phosphorylation and inactivation of the pyruvate dehydrogenase (PDH) complex. This phosphorylation cascade results in a blockade of mitochondrial pyruvate metabolism and ROS generation and an increase of glycolysis in the cytosol, and ultimately promotes cell proliferation and tumorigenesis [215] (figure 3*B.6*). In pancreatic cancer where ectopic nuclear expression of PGK has been observed, regulation of gene transcription is attributed to it. In the nucleus, PGK-1 functions as a transcriptional factor that represses E-cadherin expression and decreases redistribution of cytosolic β-catenin to the cell membrane and subsequent loss of tumour cell–cell adhesions [500]. This could indicate that nuclear PGK-1 is linked to Wnt signalling [501] and the upregulation of cell migration, synergistically contributing to the metastatic phenotype.

*ENO* is another glycolytic enzyme with several non-metabolic functions in cancer (figure 3*B.6*). Some isoforms of this enzyme, specifically ENO-1, have been identified in at least 13 cancer types. This enzyme is associated with functions related to the regulation of pathways involved in cell metabolism, proliferation and survival, which is why it has been considered a tumour marker enzyme and a target for anticancer therapy [447]. In glioma cells, ENO-1 activity is modulated by the Homer3/WBP2 complex. Interaction with this complex not only stimulates the activity of ENO-1 and subsequent promotion of the glycolytic pathway, but also gives ENO-1 the ability to activate the AMPK/AKT pathway, involved in apoptotic-resistance and autophagy [502] (figure 3*B.6*). It should be noted that one of the distinctive features of ENO-1 is that it can be externalized/secreted to contribute to modulating cell migration and the immune response. In some cells, this enzyme acts as a surface-linked plasminogen (PLG)-binding receptor and mediates the conversion of this protease precursor to active plasmin (PLN), which facilitates degradation of the extracellular matrix (ECM) and in turn enables tumour cells to invade and metastasize into other tissues [503] (figure 3*B.6*). Paradoxically, ENO-1, being externalized and exposed on the surface of the cell, is recognized by the immune system as an autoantigen, promoting the production of autoantibodies that could be favourable in the inhibition of tumour progression [504,505].

*PKM2* phosphorylation through the mTOR pathway not only drives glucose consumption but also endows this enzyme with the ability to function as a transcriptional regulator, regulating the function of HIF-1α and STAT3. In the nucleus, PKM2 can directly interact with HIF-α and enhance its binding and recruitment to p300, a transcriptional coactivator, facilitating HIF-α-dependent gene transcription. Alternatively, PKM2 may function as a kinase and activate STAT3 by transphosphorylation, stabilizing its nuclear localization [506,507] (figure 3*B.6*). The activation of these transcription factors is possibly one of the most important molecular signatures of tumour cells, since they regulate many genes involved in proliferation, survival, cellular migration, angiogenesis, inflammatory microenvironment and immunosuppression [508,509]. It has also been found that PKM2 phosphorylates β-catenin and recruits it to the CCND1 promoter, promoting the detachment of histone deacetylase 3 (HDAC3) from the promoter and consequently the acetylation of histone H3 and expression of cyclin D1 [510]. Overexpression of cyclin D1 results in the alteration of cyclin-dependent kinase (CDK) activity and rapid cell growth [511].

Moonlighting functions have also been attributed to *LDH-A* in tumour cells (figure 3*B.6*). In HPV16-positive cervical tumours, LDH-A is predominantly located in the nucleus and acts as a sensor of ROS accumulation [512]. In these cells, LDH-A converts α-ketobutyrate (α-KB) to α-hydroxybutyrate (α-HB) and induces histone H3K79 hypermethylation by DOT1L (disruptor of telomeric silencing 1-like), ultimately leading to activation of antioxidant gene expression and the Wnt signalling pathway, which contribute to maintaining cellular redox balance and cell proliferation under oxidative stress [512] (figure 3*B.6*). For its part, the upregulation and nuclear localization of *TPI* in response to chemotherapy drugs and oxidative stress induces the colony formation and migration of lung cancer cells in lung adenocarcinoma; however, the mechanism is unknown [213].

It is thus established that glycolytic enzymes play an important role in cancer, serving as a link between various signalling pathways. Any alteration in the expression or location of a glycolytic enzyme has important effects on both metabolism and gene expression, implying an effect on tumour development and progression. Resistance to chemotherapies is primarily related to qualitative or quantitative alterations in glycolytic metabolism. In this sense, these enzymes could be used as important tools for diagnosis, prognosis and treatment of various types of cancer.

### 4.3. Parasitic diseases

Parasitic diseases are a group of diseases which often have a devastating effect on health and socioeconomic levels throughout the world. Some of these diseases are included in the group of the 20 diseases considered as neglected tropical diseases (NTDs) by the WHO. These diseases, which threaten the health of millions of people, have largely been ignored by pharmaceutical industries since they occur mainly in populations in low-income countries. NTDs affect nearly two billion people, disproportionately women and children [513], and are responsible for approximately 200 000 deaths per year [514]. Existing treatments for many of these NTDs have some limitations such as lack of specificity and/or efficacy, high degree of toxicity, low bioavailability and complex application for the affected populations. All this has implied the search for new therapeutic strategies that can serve for the development of NTD treatments [515].

Some of the NTDs are caused by kinetoplastids [513]. In addition, this group of 20 NTDs contains also diseases caused not by parasites but viruses and bacteria. Other serious parasitic diseases like malaria and toxoplasmosis, also affecting many people predominantly but not exclusively in low-income countries, are not considered NTDs because considerable funding is available for their research and treatment. In these diseases, glycolytic enzymes play important roles in the life cycle and metabolism of the parasite, as well as in drug resistance, virulence and parasite–host interactions [180,516–523]. Therefore, these enzymes have been proposed as candidates for the development of diagnostics, vaccines and drugs against these diseases [524–530]. In the following sections, we will analyse the role of glycolytic enzymes in the pathogenesis of some parasitic diseases, emphasizing American trypanosomiasis (also known as Chagas disease) and toxoplasmosis.

#### 4.3.1. Chagas disease

Chagas disease (ChD) is caused by the parasitic protist *T. cruzi*. The disease is endemic in 21 countries of the Americas where the insect vector spreading it occurs, although the disease has a globalized distribution due to emigration of infected people, etc. In the American continent, it represents a serious public health problem and is one of the main causes of morbidity and mortality. This disease affects approximately 7–8 million people, mostly in rural and isolated areas, and causes 50 000 deaths per year [531,532]. Clinically, this disease develops in two phases, an acute phase after infection (which may be asymptomatic or with non-specific symptoms) and a chronic phase (which may also have a total absence of signs and symptoms of the disease), with 30–40% of patients developing multiorgan complications, mainly cardiomyopathy or mega viscera, dermatological manifestations, peripheral neuropathy and premature death [533,534].

Many studies of the role of glycolytic enzymes in the parasite have demonstrated the relevance of these enzymes in the trypanosome’s metabolism, virulence and interaction with the host [527,529,535–538]. Additionally, it has been reported that *T. cruzi* infection induces alterations in the glycolytic pathway of host cells, contributing to the pathogenesis of the disease. This latter point will be addressed in more detail in the following sections.

##### 4.3.1.1. Alterations in the inflammatory response

**Figure 4.**
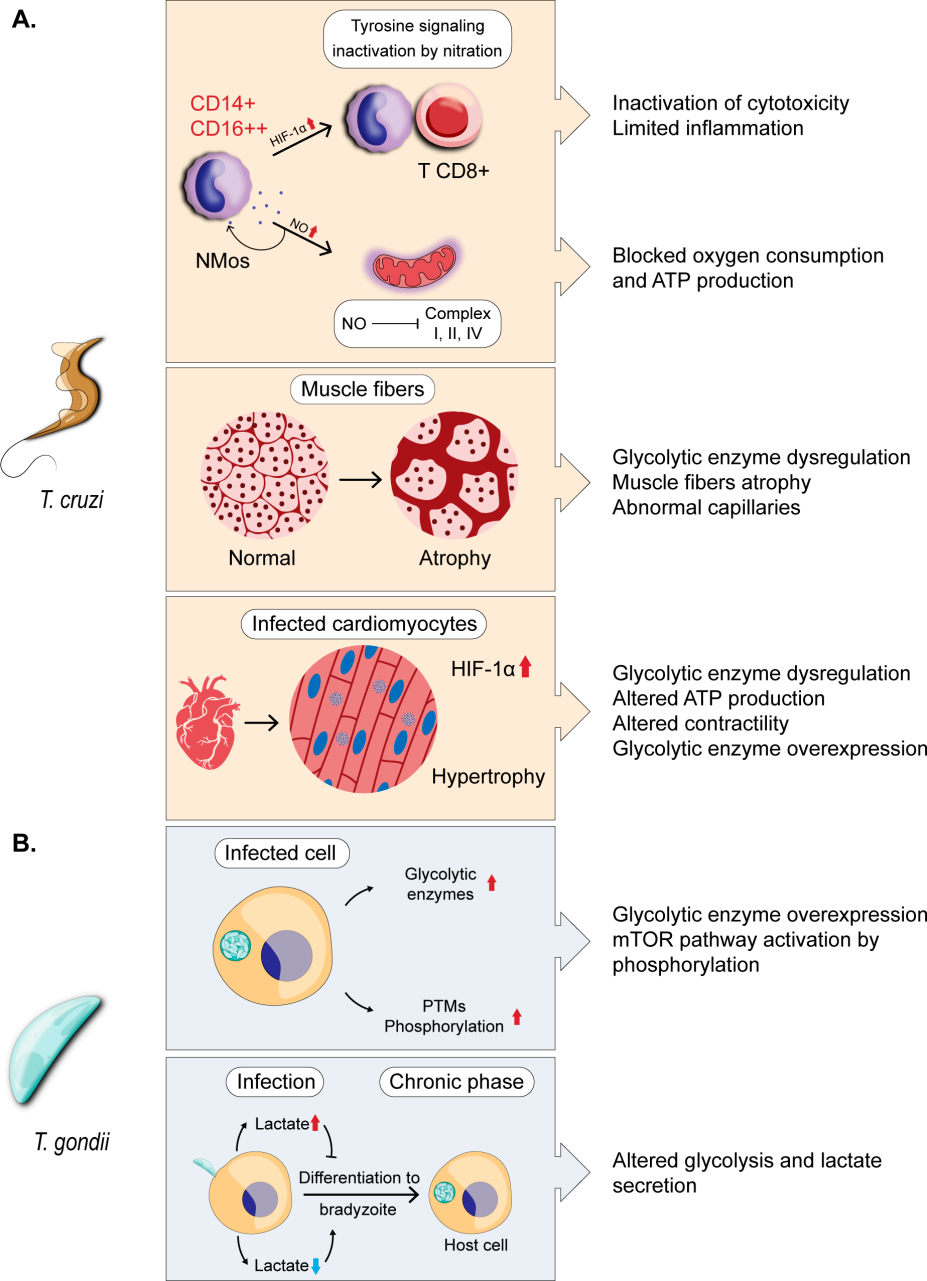
Glycolytic dysregulation effect during *T. cruzi-* and *T. gondii*-borne diseases. (*a*) Chagas disease (ChD). *T. cruzi* is responsible for inducing NMos which, among other characteristics, express HIF-1α factor and high levels of nitric oxide (NO). The high concentration of NO is responsible for inactivating tyrosine residues of signalling proteins by nitration leading to inactivation of the cytotoxicity response by CD8+T cells. On the other hand, in NMos mitochondria, NO inhibits complexes I, II and IV, thereby blocking oxygen consumption and ATP production (OXPHOS). In other cells, such as muscle cells, the contractile properties of muscles are significantly diminished during *T. cruzi* infection. Additionally, atrophy in fibres I and II, along abnormal capillaries, is observed in patients with advanced ChD. These changes are associated with an increased glycolytic flux and reduced oxidative activity. In cardiac tissue, *T. cruzi* infection induces increased expression of genes related to glycolysis, HIF-1α signalling and innate and adaptive immune responses, as well as a negative regulation of the expression of genes coding for proteins of the contractile apparatus (such as those of the sarcomere and cytoskeleton). All of this contributes to the dysfunction, cell death and progression of chagasic cardiomyopathy. (*b*) Toxoplasmosis. In *T. gondii* infection, the overexpression of glycolytic enzymes and their modulation through PTMs contribute to generating the energy requirements for the parasite to continue growing and replicating. During invasion of the host cell, the increasing lactate concentration due to glycolytic activity causes inhibition of the transformation of *T. gondii* trophozoites to bradyzoites. In this regard, it could be possible that the parasite senses target cells for invasion and chooses those with the lower lactate concentration that allows the bradyzoite differentiation process.

Glycolytic activity has been associated with proinflammatory functions of immune cells, especially during some infectious processes [18,539]. Some studies have shown the influence of glycolysis on the functionality of immune cells during *T. cruzi* infection [523]. Under hypoxic or inflammatory conditions, gene transcription is activated in immune cells such as monocytes (Mos) and is orchestrated by transcription factor HIF-1α. The accumulation of HIF-1α requires the metabolism of glucose to pyruvate to prevent its aerobic degradation. HIF-1α induces an increased expression of glucose transporters and glycolytic enzymes. An increase in glycolytic flux in conditions of hypoxia has profound effects on the cellular physiology of immune cells. Some processes affected are the modulation of immune cell activation and polarization, promotion of differentiation, as well as an increased infiltration capacity and release of cytokines [433]. Some studies have reported that infection by *T. cruzi* induces an increase in the glycolytic flux in monocytes with drives the expansion of nonclassical monocytes (NMos) (CD14+CD16++) and influences the ability of CD8+T cells to respond effectively during the chronic phase of ChD. These NMos are characterized by HIF-1α expression (the transcription factor stimulating the expression of glucose uptake, glycolytic activity, mitochondrial dysfunction and the inducible nitric oxide synthase (iNOS) leading to overproduction of nitric oxide (NO)), as well as an increased rate of production of IL-1β and IL-6 [523] (figure 4).

NO overproduction in NMos leads, through cell–cell contact, to nitration of CD8+T cell signalling proteins (NO_3_^−^ reacts with tyrosine residues of their surface proteins) resulting in their inactivation. It affects their cytotoxic function with decreased expression of the CD107a receptor (a marker of degranulation) and decreased production of proinflammatory cytokines (IFN-γ, TNF and IL-2). This process leads ultimately to dysfunction of these cells and limits inflammation [523] (figure 4) and is observed in the indeterminate phase of patients with ChD [540]. These results highlight a relevant relationship between monocyte metabolism and the functionality of the immune system in ChD. This possible modulatory role of glycolysis in Mos could be exploited therapeutically to restore or improve the immune response in patients against the parasite.

Importantly, NO production also has an autocrine effect on the monocytes themselves, since inhibition of mitochondrial electron transport may occur by the nitrosylation of the iron–sulfur-containing proteins from complex I, complex II and complex IV (cytochrome c oxidase), thereby blocking OXPHOS and increasing the production of ROS and consequently the propensity to cell death [541] (figure 4). All this could be related to structural and functional alterations of cardiac and skeletal muscle mitochondria in murine models with ChD [542,543]. Also, the products of mitochondrial damage (mitochondrial ROS production, release of mDNA into the cytosol) are common factors in the activation of the NLRP3 inflammasome [544]. Therefore, having impaired mitochondrial function, cells will enhance glycolytic metabolism to produce the ATP necessary for sustaining metabolic demands and the inflammatory state, as well as the inhibitory status of cytotoxic T cells [523]. It should be noted that alterations in the activity of mitochondrial enzymes have been detected by analysis of simple blood extracts [545], which would suggest the feasibility of using these enzymes as markers of ChD, especially the indeterminate phase of the disease. Furthermore, understanding the pathological immune mechanisms that support the inflammatory environment in ChD could be key to designing therapies.

##### 4.3.1.2. Alterations to the muscular system

##### 4.3.1.2.1. Skeletal muscle

Glycolysis is highly relevant during muscle development, ageing, physiological adaptations and skeletal muscle pathologies [546]. Alterations in the muscular system are other clinical manifestations of ChD, many of these being associated with an inflammation process, generating myositis, degeneration, atrophy and necrosis of myofibrils according to the phase of the infection [543]. Loss of muscle strength in chagasic patients has also been reported [547]. This is because skeletal muscle is considered the main site of persistence during the chronic stage when parasites are found in large meganids within muscle fibres [548]. Although only few studies focused on glycolysis and skeletal muscle disorders, an increase in glycolytic function and a decrease in the oxidative capacity of peripheral muscles of chagasic patients have been found [549] (figure 4).

The glycolytic pathway appears to have a dual role in skeletal muscle structural alterations [546]. Its upregulation can lead to either muscle hypertrophy or atrophy, which are two opposite outcomes affecting the size and function of the muscles [546,550]. Hypertrophic muscle fibres reprogramme their metabolism similarly to proliferating and tumour cells; the primary cause of muscle hypertrophy is a Warburg-like reprogrammed metabolism in which an increased glycolytic flux funnels metabolites into anabolic pathways [550] (figure 4). Notably, IGF-1/PI3K/AKT/mTOR signalling participates in this metabolic reprogramming in hypertrophic cells [550], similarly to that in tumour processes [551]. Also similar is the increased expression of some glycolytic enzymes (such as ALD, PGK and ENO) during the experimental muscle atrophy process [546]. In the case of chagasic patients, the contractile properties of muscles are significantly diminished during *T. cruzi* infection. Additionally, functional changes in this tissue are correlated with the evolution of tissue parasitism and tissue injury. During the acute phase, there is an increase in type I and a decrease in type II fibres and vice versa during the chronic phase [543,552,553]. Atrophy in fibres I and II, along abnormal capillaries, is observed in patients with advanced ChD [552]. These changes are associated with an increased glycolytic flux and reduced oxidative activity [543], as reflected by a decrease in the activity of enzymes such as LDH and the TCA-cycle component citrate synthase, and an increase in GADPH activity [552]. On this basis, glycolytic enzymes could be used as new signature molecules to improve diagnosis and may be useful for the identification of new therapeutic targets in neuromuscular disorders observed in ChD. Additionally, they may be used as biomarkers that could give useful information about bioenergetic alterations during the disease.

##### 4.3.1.2.2. Cardiac muscle

In cardiac muscle tissue, glycolysis plays an important regulatory role, mainly because the ATP produced by this pathway plays a crucial role in maintaining the contractile function of the heart. Alterations in glucose metabolism have been associated with fibrosis, apoptosis, contraction inhibition and cardiac hypertrophy [554]. However, during physiological oxygen levels, cardiac cells obtain 90% of their ATP from mitochondria through OXPHOS. Nonetheless, glycolysis is critical for normal cardiac excitation–contraction coupling (ECC; the sequence of events that links the action potential to Ca^2+^ release from the sarcoplasmic reticulum (SR-Ca^2+^) and muscle contraction), through regulation and modulation of SR-Ca^2+^ [555] by the generation of ATP and glycolytic products/intermediates that interact with the Ca^2+^ release channel (also known as the ryanodine receptor, RyR) [556].

It is known that cardiac tissue may be one of the main cellular reservoirs for *T. cruzi* [557], and infections by this parasite influence the glycolysis activation and disturbances in intracellular calcium mobilization within the heart muscle [557–560]. Experimental infections of human cardiomyocyte cell lines by *T. cruzi* revealed an increase in the expression of genes related to glycolysis, HIF-1α signalling and the innate and adaptive immune responses, and downregulation of expression of genes participating in the contractile apparatus (such as sarcomere and cytoskeleton proteins). The authors concluded that these changes contribute to dysfunction, cell death and progression of chagasic cardiomyopathy [557] (figure 4). Specifically, genes encoding glycolytic enzymes that were significantly upregulated included HK-2, HK-1, PFKP, TPI, GAPDH, PGK-1, PGM, ENO-1, ENO-2 and LDH-A, as well as other HIF-1α targets related to glucose metabolism such as GLUT1, GLUT4, PFKBP3 and vascular endothelial growth factor B (VEGFB) [557]. VEGFB regulates glucose and lipid metabolism in cardiomyocytes. Overexpression of VEGFB in cardiac cells facilitates increased glucose uptake and metabolic reprogramming that favours glucose utilization [561]. An important finding of these studies was that expression of GLUT1 and GLUT4 facilitated not only glucose uptake but also the entry of *T. cruzi* into the host cell. This suggests a manipulation of immuno-metabolic signals by *T. cruzi* to promote the entry and invasion of the parasite [557]. Also, reduced expression of genes encoding contractile proteins and activation of glycolysis would directly affect contractility and ATP production [557] (figure 4). The alteration in ATP would have a direct effect on SR-Ca^2+^ and consequently on ECC, because any disturbance that causes even subtle changes in glycolytic flux could affect the release of SR-Ca^2+^ and ECC [556,559,560], ultimately causing diastolic dysfunction (figure 4), an important hallmark of disease severity in patients with Chagas cardiomyopathy [562].

Increased glycolytic flux is generally indicative of hypertrophy of the heart [563] that accompanies various forms of cardiac dysfunction during ChD [564]. Therefore, alterations in glycolytic metabolism could be associated with cardiac cell stress during *T. cruzi* infection. The altered utilization of glucose would not only limit the anabolic capacity of the heart but also affect its ability to repair damaged tissue and detoxify free radicals [558]. Therapeutic strategies that will allow the normal metabolism of cardiomyocytes could be a great tool to attenuate *T. cruzi* infectivity, improve cardiac energy and reduce the probability of heart failure during ChD.

### 4.3.2. Toxoplasmosis

Toxoplasmosis is a zoonosis with global distribution, caused by the obligate intracellular apicomplexan parasite *T. gondii*, which affects approximately one-third of the world’s population [565]. In immunocompetent individuals, the infection is generally asymptomatic or manifests non-specific clinical signs; however, it can be severe or fatal in immunocompromised patients, as reactivation of latent infection can lead to the development of encephalitis [566]. Sometimes, infection with this parasite presents also a risk factor for the development of some types of cancer [567]. Furthermore, when a parasite is transmitted through the placenta, the life of the foetus may be compromised. In newborns, some serious symptoms (such as mental retardation and ocular disease) can be observed [565].

*T. gondii* fulfils functions related to its growth, asexual reproduction, metabolic plasticity and its invasion of host cells and egress from them [516,517,568–570]. In each nucleated host cell, *T. gondii* can, during its replication, orchestrate metabolic interactions with its host to acquire crucial nutrients and withstand metabolic demands [571]. Similar to other apicomplexan parasites [572], *T. gondii* infection induces metabolic alterations in infected cells, including changes in pathways involved in the metabolism of carbohydrates, such as glycolysis [573,574]. Changes in pathways linked to glycolysis, such as the TCA cycle and PPP, influence the transcriptomes of the parasite and/or host cell [574]. Also, changes occur in the joint *T. gondii*–host metabolome during the course of infection, especially of intermediates of several anabolic and catabolic pathways, particularly glycolysis [574]. The relevance of the alteration of some metabolic processes in the host, specifically glycolysis, during the pathogenesis of toxoplasmosis will be covered in detail in the following sections.

#### 4.3.2.1. Metabolic support of the parasite during the infection process

*T. gondii* is characterized by being highly adapted to the host and by its ability to subvert the immune system enabling it to establish a lifelong chronic infection [575]. This adaptation is mainly based on the manipulation of a wide range of cellular functions in the host. Phenotypic alterations induced by infection with this parasite are characterized by marked changes in gene expression of factors involved in the intermediary metabolism of the host target cell [573,576–580]. Infection of human foreskin fibroblasts (HFFs) by *T. gondii* induces metabolic dysregulation. It involves significant changes in the host transcriptome, some of them being the overexpression of genes linked to the metabolism of glucose and lipids, and mitochondrial respiration [573,576]. The transcriptional alteration of these metabolic genes is directly related to the regulation of HIF-1α expression, which ultimately leads to an increase in both glycolysis and mitochondrial respiration [573]. However, recent studies have reported that the nature of the metabolic reprogramming of HFF by *T. gondii* depends on the stage of infection. In the early infection stage, the host cell’s intracellular glucose concentration increases and its glycolytic, PPP and TCA-cycle enzymes are upregulated, while during late infection, gene expression of host glycolytic and PPP enzymes decreases, whereas some TCA-cycle enzymes still remain upregulated [580]. Likewise, analysis of the transcriptome of *T. gondii* infected bone marrow-derived dendritic cells (BMDCs) revealed significant alterations in mRNAs associated with cellular metabolism. Also, glucose uptake and LDH activities were upregulated in BMDC cultures infected with this parasite [579].

All these findings suggest that *T. gondii* intentionally disrupts the host’s metabolism. Being an obligate intracellular parasite, *T gondii* is dependent on the host to acquire its nutrients. This strategy would make it possible to support its metabolic demand, while avoiding both the premature death of the host due to metabolic overload, and its own elimination by the host defence system [573,580]. Notably, the expression modulation of genes for at least two metabolic processes, involving glucose and mevalonate, happens only after parasite entry/infection. The fact that this expression occurs late, because it is dependent on the direct presence of the parasite within the host cell, implies that products secreted by the parasite or infected cells are insufficient to induce changes in the expression of these metabolic genes [576].

Proteomic analysis of HFFs after infection by *T. gondii* detected in total 157 proteins of which levels were significantly altered, with glycolytic enzymes representing 13% of these proteins. Among these glycolytic enzymes are ALD-A/B, ENO-1, GAPDH, PGK-1, PKM2 and TPI, most of them being upregulated [574,577] (figure 4). The genes of these glycolytic enzymes, which together with apoptotic genes are modulated during infection, are considered ‘*proparasitic genes*’, that is, host genes required for parasite survival. Increased glycolysis results in increased production of both ATP and pyruvate, which directly or indirectly enter various biosynthetic pathways in the cell, as well as fuelling OXPHOS. All this would result in the metabolic reprogramming of the host cell [576].

It has been documented that *T. gondii* infections induce alterations in host cell proteins, many of these changes being the result of PTMs, of which phosphorylation turned out to be one of the most important modifiers [581,582] (figure 4). A total of 1796 and 590 phosphopeptides and phosphoproteins, respectively, were identified in HFF cells infected with *T. gondii*. Most of these phosphoproteins, close to 80%, were located predominantly in the cytosol and nucleus. Approximately 21% of the phospho motifs and several pathways, such as glycolysis/gluconeogenesis, as well as the host cell’s mTOR pathway, were observed to be enriched during infection by the parasite (figure 4). In the case of the mTOR pathway, both proteins that play roles in the regulation of the pathway’s core (e.g. AMPK-1, PDK-1 and TSC-2) and proteins that are substrates of the core pathway (such as EIF4EBP1, RPS6 and EIF4B), were included within the group of phosphoproteins. This suggests that *T. gondii* infection may cause phosphorylation of host mTOR substrates and selectively promote the translation of certain mRNAs [581]. Notably, these analyses also showed enrichment of many phosphopeptides derived from glycolytic enzymes in HFF cells after infection with *T. gondii*. Like overexpression, modulation of glycolytic enzyme activity by PTMs would allow the high energy production required to meet the demand of fast growing and multiplying tachyzoites; both processes are critically involved in the expenditure of cellular energy [581].

#### 4.3.2.2. Parasite life cycle progression

The impact of host cell physiology on the progression of the *T. gondii* life cycle has been determined [583]. A transition from acute primary infection to chronic toxoplasmosis is accompanied by a change in the development from rapidly replicating, metabolically highly active tachyzoites to largely dormant, slowly replicating bradyzoites within tissue cysts. This differentiation process is critical for the parasite to complete its life cycle and for pathogenesis. However, this differentiation exhibits a clear tissue tropism and may be influenced by the metabolism of the host cell [584]. When performing *in vitro* assays to study bradyzoite conversion, it was found that different cell types vary significantly in their ability to facilitate this differentiation. In some cell lines, such as NIH3T3 and HEK293 cells, inhibition of conversion to bradyzoites (conversion-resistant phenotypes) was observed, while other cell lines, such HFF and Vero cells, were more permissive (conversion-permissive phenotypes) for the transformation from tachyzoites to bradyzoites. The main difference between both cellular phenotypes is their glycolytic activity and production of soluble factors, such as low-molecular-weight metabolites including lactate [584]. The data indicate that host cells with high production levels of lactate promote tachyzoite growth over bradyzoite conversion.

It is possible that after proliferation within the infected cell, the parasite may have the ability to ‘sense’ the cellular microenvironment, disseminate within its host and initiate its bradyzoite differentiation process inside different cells. This could explain the predominant localization of the parasite in the neural and muscular tissues during chronic toxoplasmosis [583] (figure 4). Alternatively, the tropism for some cell types could be related to the fact that non-proliferating host cells, like neurons and skeletal muscle cells, can express an effector or provide a stressful environment that stimulates differentiation into a bradyzoite, characterized by slow growth. In contrast, proliferating host cells, which by maintaining a glycolytic metabolic profile, with high turnover of glycolytic intermediates and high rates of lactate production, can support the parasites’ biosynthetic activities [571].

## 5. Conclusion and possible trends for future research

Glycolysis is a metabolic pathway that is present in almost all organisms. This process of glucose catabolism is carried out in 10 successive chemical reactions, each catalysed by a specific enzyme. In addition to contributing to cellular metabolic support, through the generation of ATP, NADH and intermediate metabolites, glycolysis plays various important roles in the development of some neurodegenerative and parasitic diseases of humans and cancer. Alterations in the subcellular location, activity and expression of the enzymes that participate in this pathway appear to be involved in the development and progression of these pathologies. Therefore, studying glycolytic enzymes in these diseases is of great relevance to improving prognostic, diagnostic and therapeutic strategies for all these diseases, for which until now no effective treatment is available.

## Data Availability

This article has no additional data.
